# A Systematic Review of Evidence on the Role of Ready-to-Eat Cereals in Diet and Non-Communicable Disease Prevention

**DOI:** 10.3390/nu17101680

**Published:** 2025-05-15

**Authors:** E. J. Derbyshire, C. H. S. Ruxton

**Affiliations:** 1 Nutritional Insight, Epsom KT17 2AA, UK; 2 Nutrition Communications, Cupar KY15 4HQ, UK; carrie@nutrition-communications.com

**Keywords:** breakfast cereal, diet, dietary shortfalls, health, micronutrients, nutrient density, ready-to-eat cereals, ultra-processed foods

## Abstract

**Background:** Ready-to-eat cereals (RTECs) are a large, heterogeneous category of cereals designed to fit into busy lifestyles with minimal preparation time. **Methods:** This systematic review evaluated nutrient intake data from seven national surveys. Using PubMed and Science Direct (1 January 2004 until 16 September 2024), we investigated RTECs in relation to their contributions to macro, micronutrient and food group intakes, breakfast/diet quality and effects on health with focus on non-communicable disease (NCD) prevention. The search was restricted to Systematic Reviews (SRs), meta-analyses (MAs), randomised controlled trials (RCTs) and observational studies. Fifty-one publications were obtained. Studies related to health outcomes and NCD risk were graded using an updated Scottish Intercollegiate Guidelines Network approach. **Results:** Grade A evidence: Based on high-quality MA, SRs, or RCTs, this showed that RTEC consumption was associated with improved nutrient intakes (particularly fibre and micronutrients), reduced cardiovascular disease and mortality. One good-quality Grade A meta-analysis showed that total whole grain intake which included cereals was associated with a reduced risk of total cancer. Grade B evidence: Based largely on observational evidence, this showed that RTEC consumption was associated with reduced risk of overweight and obesity, body mass index and composition improvements and type 2 diabetes risk. For food group intakes, breakfast/diet quality and lipid profiles, more well-designed studies were needed (Grade D evidence). **Conclusions:** There is consistent evidence that RTECs generally have positive or neutral effects on nutritional status and NCD prevention. Strongest evidence exists for RTEC and micronutrient intakes, reduced risk of cardiovascular diseases (CVDs), body weight regulation, and reduced type 2 diabetes risk. Public health messaging should recognise that RTECs, especially whole-grain, higher-fibre and lower-sugar varieties, may help to reinforce micronutrient intakes and a range of health outcomes.

## 1. Introduction

RTECs are a large, heterogenous category comprising a variety of cereals with varying nutritional profiles [1]. They are processed grain formulations made predominantly from wheat, corn or oats that are suitable for human consumption without further cooking [2]. Cereals provide the majority of the global population’s energy intake and have been consumed by humans for approximately 100,000 years [3,4]. They can make a valuable contribution to nutrient intakes, including fibre, whole grains, vitamins (B vitamins, vitamins A and D) and minerals (calcium, magnesium, iron, zinc, phosphorus and potassium) and are regarded as an affordable breakfast option [5].

RTECs require a minimal amount of processing to improve digestibility, nutrient availability, and palatability [6]. Various RTECs are produced through methods such as flaking, steaming, rolling, puffing, extrusion, shredding whole grain and baking [7]. Whole grains are processed kernels from which only the inedible parts have been removed [8]. The processing of cereals enhances shelf-life, stability, functional properties, convenience, and reduces waste [9,10,11]. It also addresses quality and safety issues caused by microorganisms like mould mycotoxins [12]. Additionally, processing improves the bioavailability and digestibility of nutrients while reducing non-nutrients like phytic acid [13]. Fortification can replenish vitamins and minerals lost during processing and improve micronutrient intake in children and adults [14].

The World Health Organisation defines NCDs as chronic diseases that tend to have a long duration and are the result of a combination of behavioural, environmental, genetic and physiological factors; diabetes, cancers and cardiovascular diseases (CVDs) are some of the main NCDs that can be dietary-related [15]. NCDs were attributed to the deaths of 43 million people globally in 2021, which was equivalent to 75% of deaths that were not pandemic-related [15]. Within the EU, the economic burden of NCDs is rising, with CVDs costing EU healthcare systems around EUR 111 billion and cancer costing EUR 97 billion [16,17,18]. In 2019, estimations found that overweight and obesity cost Europe around EUR 464 billion (≈EUR 141 billion as direct costs and EUR 323 billion through indirect costs) [19].

Changes in lifestyle with respect to diet and nutrition have been identified as one of the most effective preventative strategies for NCDs [20]. Data on dietary risk factors from the 2019 Global Burden of Disease (GBD) Study on deaths and disability-adjusted life-years (DALYs) found that 7.9 million deaths and 187.7 million DALYs were diet-related, with a low intake of whole grains being one leading dietary risk factor [21]. In the USA, Canada, Europe and Australia, RTECs are an important component of breakfast, with whole-grain, fibre-rich or fortified options being linked to favourable nutritional (micronutrient profiles) and health outcomes including beneficial effects on type 2 diabetes and hypertension [1]. Yet recently, potential health benefits of RTECs and roles in NCD prevention are beginning to be tarnished by growing attention on the degree of food processing [22,23]. Yet, for certain subgroups of processed foods such as cereals, breads and plant-based foods, the risks of NCDs such as cardiometabolic diseases and cancer have not been identified [24]. Two previous systematic reviews published in 2014 and 2016 related to RTECs and key nutritional and health outcomes found promising roles in relation to reduced risk of CVD, diabetes, overweight and obesity [1,25]. Since those reviews, numerous meta-analyses and systematic reviews have been published that examine the relationship between breakfast cereal consumption and disease risk, many of which are framed within the context of the NOVA classification of processed foods.

The aim of the present review is to examine latest evidence related to RTECs in relation to their contributions to daily macro- and micronutrient intakes, food group intakes, breakfast/diet quality, nutrient intakes/status and implications for NCD prevention. Studies that investigated subgroups of ultra-processed foods (UPFs) as defined by NOVA classifications including breakfast cereals, are discussed, given increasing interest in this topic.

## 2. Methods

### 2.1. Nutrient Intakes from National Surveys

To first identify RTEC contributions to macro- and micronutrient intakes, an evaluation of data from national diet, food and health surveys was undertaken. This included data from Canada, France, Ireland, the United Kingdom and the United States of America as these countries have established and publicly available food/nutrition and health survey datasets. Furthermore, these countries have a tradition of consuming breakfast cereals for breakfast. The range of nutrients extrapolated was based on those most consistently reported in the surveys and present in RTEC. This formed the first part of the results.

### 2.2. Systematic Review Search Strategy

Studies published from 1 January 2004 to 16 September 2024 were evaluated. PubMed was searched to capture publications related to RTECs and the diet and health of children and adults. Science Direct was also searched to identify additional publications, excluding conference abstracts and replica publications. In many studies, RTECs are referred to as ‘breakfast cereals’; hence, both of these terms formed the basis of the search terms [26].

The search terms “ready-to-eat cereal”, “RTE cereal”, “RTEC”, or “breakfast cereals” were used. The outcome terms included “modelling”, “food groups”, “breakfast quality”, “diet quality”, “nutrient intakes”, “nutritional adequacy”, “micronutrient”, “glucose levels”, “lipid” and in relation to NCDs “body weight”, “type 2 diabetes (or T2D)”, “cardiovascular disease (or CVD)” and “cancer” (as per the WHO definition of NCDs [15]), along with “ultra process*”, given the growing interest in levels of food processing and ultra-processed foods (UPFs). A full list of search terms is included in Appendix A. From the initial search, titles and abstracts were first reviewed and publications identified as candidates for full-text screening. Publication reference lists were also screened to identify additional publications.

### 2.3. Inclusion/Exclusion Criterion

Inclusion criteria were as follows: Global publications written in the English language, studies conducted in children and adults of both sexes and all age groups, except for colorectal cancer, for which an age restriction of 19+ years was applied, due to age being an established risk factor [27]. The search was restricted to systematic reviews (SRs) and meta-analysis (MAs) publications, randomised controlled trials (RCTs) and observational studies which included individual prospective cohort studies (PCSs) and cross-sectional (CSs) studies. For the health outcomes, due to the large body of available publications, the search was restricted to SRs and MAs only. Studies were excluded if they did not meet these specifications.

**Figure 1:**
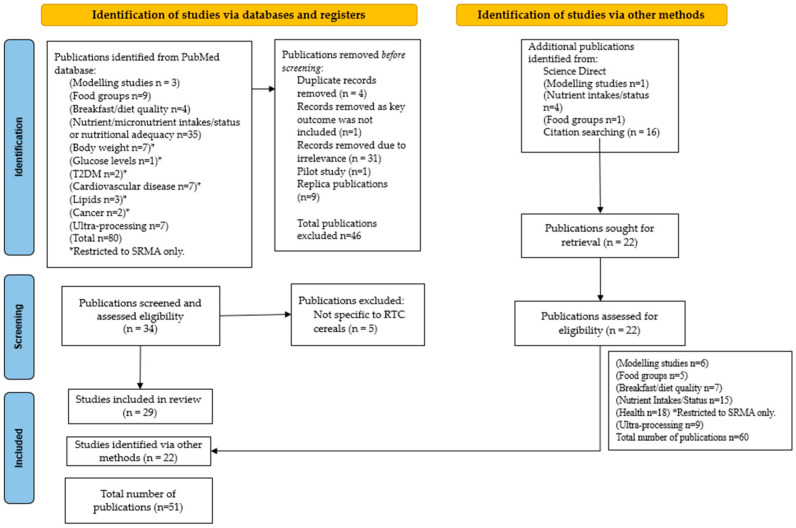
PRISMA 2020 flow diagram for reviews which included searches of databases and other sources. Adapted from the work of Page et al. (2021) [28]. * Restricted to SRMA only.

Figure 1 shows the PRISMA study selection flow diagram for included searches of databases and other sources [28]. Author ED identified key publications for inclusion within the review, and author CR undertook a cross-check.

### 2.4. Data Extraction

Studies were presented in tables according to the hierarchy of evidence. Therefore, SR and MA publications were listed first, followed by RCTs and observational studies. The typical hierarchy of evidence pyramids classifies research according to study type, but not all the evidence placed at the same level has the same quality [29]. The updated Scottish Intercollegiate Guidelines Network scale [29] considers levels of study bias and was applied to grade the quality of evidence for publications related to health outcomes (Table 1 and Table 2). Standardised tables were used to extract information from systematic reviews, meta-analysis and key studies which included the following: author, year, PubMed ID (PMID), country of residence of the participants, study design, number of studies included in the publication and the number of participants, outcomes and results (Appendix B.1, Appendix B.2, Appendix B.3, Appendix B.4, Appendix B.5 and Appendix B.6).

## 3. Results

### 3.1. Contribution to Nutrient Intakes

As shown in Table 3 and Table 4, seven national diet, food and health surveys considered the contribution of RTECs to macronutrient and micronutrient intakes. These analysed data from five different countries (UK, Ireland, France, Canada and the USA). Before evaluating these, it is important to consider that reported dietary data may reflect whole populations (including consumers and non-consumers of RTECs) or consumers only. Ideally both types of data are reported in national surveys, but this is not always the case. Both approaches are useful when formulating public health policies as these are directed to whole populations or sub-groups. Equally, it can be useful to ‘focus in’ on consumers of specific food categories to determine how these contribute specifically to nutrient intakes. As seen in the survey data, the percentage of RTEC consumers ranged from 13% (adult females in France) to 85% (Irish children) [30,31]. In general, children and adolescents were higher consumers of RTECs (29–85%) than adults or older adults (13–44%) [30,31,32,33].

Focusing on macronutrients in total populations (consumers and non-consumers), RTECs provided 1–10% of average daily energy intakes and negligible amounts of total and saturated fat (1–4%). RTECs provided 0–6% of mean daily protein intake and useful amounts of dietary fibre which ranged between 1 and 23% [30,33,34,35]. Amongst consumers, RTECs contributed to 16–19% of dietary fibre intake for children and young people aged 2–18 years [32,36,37] and around one-fifth (22–23%) of daily fibre intakes in adults aged 19 years and over [32,36,38]. Regarding sugar intakes amongst RTEC consumers and non-consumers, the percentage of the contribution of total sugars and free sugars to daily total intakes ranged from 2 to 11% for both (Table 3). For children and young people, RTECs contributed to around 8–11% of total sugars (11% of free sugars in Ireland), or 18% added sugars as derived from the United States National Health and Nutrition Examination Survey (US NHANES) data [32,33,36,37]. In the United Kingdom National Diet and Nutrition Survey (UK NDNS), the contribution of RTECs to daily intakes of free sugars was around 2–4% for the overall population [34].

In relation to micronutrients, RTECs typically contributed to daily intakes of vitamin A, folate, B group vitamins, vitamin D, calcium, magnesium, potassium, iron and zinc. Amongst RTEC consumers for children and young people, RTECs provided 32–51% of daily iron intake, 23–30% vitamin B_1_, 17–43% vitamin B_6_, 6–21% vitamin D, 9–33% zinc and 12–14% folate (as DFEs) [32,37]. Amongst adult consumers (18/19 years+), RTECs contributed to 31–49% daily iron, 13–41% vitamin B_6_, 29–33% vitamin B_1_, 10–30% zinc, 12–16% folate (as DFEs) and 3–21% vitamin D intake [32,36,37]. Among consumers and non-consumers, the contributions were still high, with RTECs providing 1–23% vitamin D, 3–28% iron, 3–22% vitamin B_2_, 2–25% folate, 1–11% magnesium, 1–8% calcium, 1–9% zinc and 1–5% potassium intake (Table 4).

In summary, RTECs contribute negligible amounts of fat/saturated fat to the diet; amongst consumers, they provide around 8–11% of total sugars and 16–23% of daily fibre. RTECs also provide an array of vitamins and minerals, including folic acid, iron, vitamin B12 and vitamin D.

### 3.2. Modelling Studies

Six key modelling studies [41,42,43,44,45,46] focused on RTECs or breakfast cereals, as shown in Appendix B.1. Mathematical modelling studies are defined as “mathematical frameworks representing variables and their interrelationships to describe observed phenomena or predict future events” [47]. Four modelling studies focused on nutrient intakes [41,42,44,45], one nutrient adequacy [42], and one vitamin D status [43].

Tucker et al. (2024), using data from children (2–18 years) from NHANES 2011-18, modelled the effects of replacing RTECs at 10, 25, 50 and 100% with a weighted composite of all other breakfasts and found that 100% RTEC replacement led to a 5% reduction in added sugars, whilst fibre decreased by 16%, vitamin D by 19% and calcium by 5% [46]. Other research based on Australian National data [41] modelled the effects of substituting grain-based UPFs with non-UPF alternatives, finding that their exclusion (which included breakfast cereals) led to significant reductions (*p* < 0.05) in folate, thiamine and iodine, indicating potential unintended consequences for vulnerable groups such as women of childbearing age. The US NHANES modelling analysis [44] investigated the effects of three RTEC fortification scenarios: (1) baseline, which was as reported in dietary surveys; (2) zero micronutrients from RTEC fortification; and (3) optimised, which were fortification levels that reduced the percentage of the population below the estimated average requirement or above the Upper Limit. Results showed that RTEC fortification can be optimised to provide key nutrients (such as B vitamins, calcium and iron) whilst reducing the percentage of population below the estimated average requirement, or above the Upper Limit [44]. Other NHANES research [45] applied model 1 (replacing American breakfast foods with RTECs) or model 2 (replacing breakfast foods with RTECs and milk), finding that model 1 diets provided significantly more folic acid (+104.6%), whole grains (+84.6%), iron (+54.5%), fibre (+14.3%), vitamin D (+14.0%), sugar (+5.0%) and a decline in solid fats (−10.9%), whilst model 2 diets were significantly higher in calcium (+11.3%) and potassium (+3.95%).

Regarding nutrient adequacy, a further US NHANES modelling analysis [42] investigated theoretical removal of different dietary patterns, which included 25, 50 and 100% removal of bread made using yeast and RTECs. The removal of yeast bread and RTECs increased the percentage of adults below the estimated average requirement for calcium, magnesium, vitamin A, C and E [42]. Folate intakes (measured as dietary folate equivalents) were 109 µg lower, iron 2.7 mg/d lower and magnesium 19 mg/d lower when breads and RTECs were 100% removed compared with the 25% removal level [42]. Finally, in the UK, a mathematical modelling analysis [43] using NDNS data (correcting for ultra-violet exposure and age) studied the effect of fortifying RTECs with 4.2 μg vitamin D per 100 g. Findings showed that RTEC fortification could raise serum 25-hydroxy vitamin D levels across all age and gender groups, particularly in males and females aged 65 years and over (by 6.98 and 5.55 nmol/L, respectively) [43].

Overall, modelling studies show that RTEC reduction or removal could have unintended consequences at the population level by lowering intakes of key nutrients such as fibre, whole grains, vitamin D, calcium, folic acid, iron (especially in girls and women) and iodine, which are already insufficient in the diets of a significant proportion of the population [48,49,50,51,52]. Results further show that tailored RTEC fortification strategies could help to ‘balance out’ habitual macronutrient and micronutrient intakes, lessening dietary gaps whilst preventing a surplus of certain nutrients.

### 3.3. Contribution to Food Groups

Five key studies [5,37,53,54,55] investigated RTECs/breakfast cereals in relation to their contribution to dietary food groups, as shown in Appendix B.2. Three publications used US NHANES data [5,37,53], one used data from the third US School Nutrition Dietary Assessment Study [55], and Michels et al. (2015) [54] collated data from n = 1215 adolescents participating in the Healthy Lifestyle in Europe by Nutrition in Adolescence (HELENA) study.

US NHANES 2015-18 data analysis [53] showed that amongst children (2–18 years), RTEC consumption was positively associated with increased intakes of recommended food groups, including whole grain and dairy intakes (*p* < 0.001). Amongst adults (19 years+), RTEC intake was also positively associated with whole grain, dairy, and fruit intake (*p* < 0.001) [53]. Research using 2017-18 US NHANES data showed that RTEC consumers (12% adults and 28% children) had higher whole grain and total dairy intakes compared to RTEC non-consumers or those not eating breakfast [5]. In an earlier US NHANES analysis (2015–16), children (0.5–17 years) who ate RTECs had a 61% higher whole grain intake (*p* < 0.0001; whole grain intake in RTEC consumers provided 48% of all whole grain intake) and 29% higher total dairy intake (*p* < 0.0001) [37].

The third School National Dietary Assessment Study in the US found that students taking part in the School Breakfast Programme who ate RTECs at breakfast obtained significantly more daily whole grains (0.71 oz. equiv.) than pupils eating a noncereal breakfast (0.43 oz. equiv.) [55]. Michels et al. (2015) [54] used data from the HELENA study (n = 1215, 12.5–17.5 years) and found that RTEC consumers ate fruit (57 vs. 51%) and milk/yoghurt (81.2 vs. 56%) more frequently, implying healthier dietary patterns.

In summary, these findings suggest that integrating RTECs into daily diets helps to increase intakes of recommended food groups, especially whole grain intakes.

### 3.4. Breakfast and Diet Quality

Seven publications investigated RTE/breakfast cereal in relation to breakfast or diet quality, as shown in Appendix B.3. Priebe and McMonagle (2016) [1] undertook a systematic review, finding that RTEC consumption (≥5 servings/weeks) in children and adults was associated with a healthier eating pattern which included more dietary fibre and less total fat. In addition, RTEC consumers were more likely to have dietary intakes aligned with recommendations.

In a US study [56] which was a 1-day randomly assigned experimental design, 91 children (5–12 years) during a camp visit were allocated to one of three groups: high-sugar cereals, low-sugar cereals, or fruit/milk/juice. The consumption of high-sugar cereals increased total sugar intake and reduced breakfast nutritional quality. In contrast, the consumption of low-sugar cereals improved the nutritional quality of breakfast, especially as fruit was added for natural sweetness [56].

Two studies were undertaken in France [57,58]. Findings from both the INCA3 and French CAFF (a cross-sectional survey) showed that a breakfast pattern including RTECs provided more fibre and micronutrients, and resulted in higher breakfast quality scores [57] or Nutrient-Rich Food Index scores compared to other breakfast patterns [58]. In Europe, the multi-centre HELENA study [54] showed that daily and frequent RTEC consumers had a significantly higher Diet Quality Index (*p* = 0.003 and *p* = 0.016, respectively) than non-consumers, with this index considering dietary quality, dietary diversity, dietary equilibrium and a meal index. Two publications [37,53] used data from the US NHANES survey with both finding that RTEC consumption was associated with better diet quality, as determined by the Healthy Eating Index-2015.

In summary, these consistent findings suggest that RTEC consumption is associated with the improvement of both breakfast quality and diet quality. Their consumption appears to correspond to healthier eating patterns, improved intakes of fibre, vitamins and minerals, and diets that are more closely aligned with recommendations.

### 3.5. Nutrient Intakes

A total of 15 key publications evaluated RTECs/breakfast cereal consumption in relation to nutrient intakes, as shown in Appendix B.4. Four systematic reviews were undertaken in this field [1,25,59,60]. Regarding macronutrients, child and adolescent RTEC consumers had a significantly higher fibre intake than breakfast skippers (mean difference; MD, −6.67; 95% CI: −11.02, −2.32), a higher daily energy intake (MD, −7.00; 95% CI: −11.51, −2.49) and lower fat intake (MD, 11.10; 95% CI: 7.15, 15.04) [59]. Similarly, for micronutrients, children and adolescents eating RTECs had significantly higher daily intakes of thiamine, vitamin B2, vitamin A, C, D, calcium, iron, magnesium and potassium than those skipping breakfast, with no trends observed for sodium [60]. Priebe and McMonagle (2016) [1] similarly found that amongst children and adults, frequent RTEC consumers (>5 times weekly) had a lower risk of micronutrient inadequacies for vitamin A, calcium, folate, vitamin B6, magnesium and zinc compared with non-consumers. A systematic review of 11 intervention trials found that childhood and adolescent RTEC consumers had daily diets with a higher percentage of energy for carbohydrate, total sugars, dietary fibre and vitamins and minerals [25].

Four trials/intervention studies have been published [61,62,63,64]. A randomised, controlled 2-week two-arm trial [61] providing overweight/obese females (18–44 y) with 30 g low-fat RTECs and 120 mL skimmed milk as a replacement for two meals daily over two weeks observed significantly higher increases in fibre, sugar, vitamin A, riboflavin, niacin, vitamin B6, and vitamin B12, and reductions in total and polyunsaturated fats and sodium intakes, compared with the control arm (*p* < 0.05). A longer 12-week RCT allocated 73 girls (16–19 y) to consume 50 g fortified or unfortified cereal with 150 mL semi-skimmed milk. There were greater increases in vitamins B1, B2, B6, B12, folate and iron (*p* < 0.001) and of vitamin D (*p* = 0.007), and biomarkers for status also improved for vitamins B2, B12, folate and iron in the fortified RTEC group compared with those eating unfortified cereal [62]. Amongst 67 overweight/obese women (20–35 y), increased consumption of breakfast cereals (diet C; consumed a minimum of three times daily) over 6 weeks led to significant improvements in folate status, which could have wider implications for neural tube defect prevention [63]. Albertson et al. (2009) randomised children (8–10 y) into a modified total/saturated fat diet or usual diet intervention, and found that after 7.5 years follow-up, RTEC was associated with all nutrient measures, indicating potential nutritional benefits [64].

Five observational studies further focused on nutrient intakes from RTECs [53,54,55,65,66]. There was a consensus that RTEC consumption contributed to more favourable micronutrient profiles. Zhu et al. (2021) [66], using 2017-18 US NHANES data, found that RTEC consumption corresponded to significantly higher intakes of calcium, iron, zinc, magnesium, potassium, phosphorus, vitamin A, B6, B12, thiamine, riboflavin, niacin, folate, and vitamin D (all *p* < 0.05). Earlier work [67] using data from US NHANES 2013–14 found that RTEC consumption in children and adults was associated with significantly improved serum 25-hydroxyvitamin D levels compared to those not eating these. Other research using US NHANES data (2013–16) [68] found that RTEC consumption amongst those taking part in the Special Supplemental Nutrition Programme for Women, Infants and Children (WIC) significantly improved intakes of calcium, iron, zinc, vitamin A, thiamine, riboflavin, niacin, B6, B12, folate and vitamin D amongst the children aged 1 to 5 years. Affenito et al. (2013) [55] found that children aged 5–18 years taking part in School Breakfast Programmes and eating RTECs had better fibre, vitamin A and iron profiles compared with those consuming a noncereal breakfast, indicating the importance of such initiatives.

Taken together, there is a strong and established (Grade A) body of evidence that RTEC consumption can help to improve the amount and array of nutrients consumed daily and reduce the risk of dietary shortfalls.

### 3.6. NCDs and Markers of Health

Nineteen SR/MA review studies investigated inter-relationships between RTECs/breakfast cereals, specified NCDs and related aspects of health (Appendix B.5).

#### 3.6.1. Body Weight and Composition

Six SRs [1,25,26,69,70] examined RTECs and outcomes related to body weight, with one including a meta-analysis [71]. Of these, three focused on children and teenagers [26,70,71]. All reported reductions in body mass index (BMI) with RTEC consumption [26,70,71].

De la Hunty et al. (2013) [71] collated data from two prospective cohorts and 11 cross-sectional studies, finding that children and adolescents regularly eating breakfast cereal had a significantly lower mean BMI and were less likely to be overweight/obese than those eating breakfast cereals infrequently or not at all. The risk of overweight was reduced by at least 10% and possibly by up to 50% in those eating breakfast cereals regularly, while mean BMI was reduced by around 1 kg/m^2^ [71]. One SR found that 14 out of 20 observational studies linked RTEC consumption with reduced BMI, odds of overweight/obesity and improved abdominal profiles than less frequent or non-consumers [70]. Data from controlled trials, however, were lacking, and bias was a potential issue, although one study reported a loss of 0.9 kg when overweight/obese children ate RTECs alongside receiving nutrition education [70]. The third publication focusing on children and teenagers was less rigorous [26]. It reviewed evidence from 12 studies (8 were cross-sectional) and found trends towards reduced BMI and weight gain, although the evidence was not graded or weighed [26].

Four SR publications drew conclusions relevant to adults, with these reporting inverse relationships between RTEC consumption and BMI/prevalence of overweight/obesity [1,25,26,69]. In particular, Priebe and McMonagle (2016) identified from seven RCTs that RTECs were higher in dietary fibre, reduced hunger, and appeared to be more satiating compared to controls [1]. Two publications concluded that RTECs could be used as a meal or snack replacement as part of weight-management programmes [26,69]. Data extrapolated from 14 RCTs suggested that RTECs could also be used as a meal/snack replacement as part of a hypocaloric diet [69].

Overall, regarding the quality of evidence, four publications [26,69,70,71] were categorised as Ma1+ or Rs1+ (well conducted with a low risk of bias), whilst two [1,25] were graded as Rs1− (systematic reviews regarded as having a high risk of bias). Given this, the overarching quality of evidence related to RTECs and body weight/profile improvements was assessed as Grade B [29].

#### 3.6.2. Type 2 Diabetes

Four publications, one MA [72] and three SRs [1,25,73], evaluated inter-relationships between RTECs and T2D. Aune et al. (2013) [72] analysed data from three cohort studies, finding inverse associations between whole-grain breakfast cereal consumption and T2D risk (RR 0.72 95% CI 0.55–0.93, *p* = 0.01). The strength of findings in this publication may have been somewhat limited due to included studies not correcting for measurement error, although there was no asymmetry observed in funnel plots [72].

Regarding the three SRs, Chen et al. (2023) collated and analysed data from three of the largest U.S. cohort studies [73]. It was found that cereals (defined as cold cereals) were associated with a lower T2D risk (HR 0.78, 95% CI 0.75–0.82), which was stronger than reductions for dark breads and whole-grain breads (HR 0.96, 95% CI 0.94–0.98) [73]. Two SRs found that the whole grain and/or fibre component of RTECs was associated with the observed lower risk of T2D [1,25]. It should be considered that observational studies typically use self-reported data, which may influence the quality of the findings [1]. Nevertheless, Williams et al. (2014) [25] allocated a Grade B level of evidence to the statement that ‘whole grain or high-fibre breakfast cereals are associated with a lower risk of diabetes.

After applying benchmarks developed by Arieta-Miranda et al. (2022) [29], the evidence for lower risk of T2D was regarded as being Grade B, due to the need for larger meta-analysis publications that collate evidence from a greater number of studies.

#### 3.6.3. Cardiovascular Disease and Lipid Levels

A large body of evidence has considered links between RTECs and CVDs, as well as markers of blood lipids. Three MA publications focused on CVDs [74,75,76]. In the SRs and dose–response MAs conducted by Sun et al. (2022), the included studies were mostly high-quality according to the Newcastle-Ottawa Assessment Scale for cohort studies [74]. Aune et al. (2016) used the same quality rating scale with the mean quality scores for CVDs and CHDs being 7.7 out of a maximum of 9 points [76]. Kwok et al. (2020) reported that many analyses had the lowest or most limited (level 4) evidence due to there being fewer than four studies [75].

Overall, from the three meta-analysis publications, there was a consensus that breakfast cereals were inversely associated with CVDs (and CHDs), indicating protective associations [74,75,76].

Turning to evidence from the SRs, there was a general agreement that RTECs, particularly those that were high-fibre or whole-grain, had the potential to lower CVD risk and favourably modify lipid profiles, particularly in men with elevated cholesterol levels [1,25,77,78]. Three SRs evaluated RTEC consumption in relation to blood lipid levels [1,25,77]. One SR using results from three prospective cohorts found that RTECs providing soluble fibre reduced low-density lipoprotein cholesterol in men with hypercholesterolemia [1]. Fortified RTECs providing folate were associated with lower plasma homocysteine levels [1]. Of the 14 studies evaluated by Beserra et al. (2020) [77], 2 studies [64,79] found that increased RTEC intake was associated with reductions in total cholesterol and low-density lipoprotein cholesterol. Williams et al. (2014) [25] graded evidence using recommendations advised by the Australian national Health and Medical Research Council, concluding that regular consumption of oat-, barley- or psyllium-based breakfast cereals can help lower total and LDL cholesterol concentrations (evidence grade A), and wholegrain and high-fibre breakfast cereals are associated with a lower risk of cardiovascular disease (Grade C; provides such support but care should be taken in its applications).

Now, a decade on, with the emergence of new, consistent meta-analytical research, links between RTECs and the reduced risk of CVDs could be regarded as Grade A for whole grain varieties using the Scottish Intercollegiate Guidelines Network criterion [29]. Continued analyses of large datasets are needed.

#### 3.6.4. Cancer

One good-quality SR and dose–response MA investigated whole grain consumption, which included those derived from breakfast cereals, finding that it was associated with reduced total cancer risk [76]. The relative risk for total cancer was 0.83 (0.77 to 0.90; I^2^ = 83%, n = 11) per 90 g/day increase in whole grain intake (equivalent to three servings, e.g., one bowl of cereal and two pieces of bread) [76]. This study was particularly useful in that it quantified potential levels of intake that could have desired health effects. The single SR/MA identified [76] was regarded as high-quality (mean quality score for the studies on total cancer was 7.8 out of 9 points), and was thus assigned Grade A level evidence according to updated approaches using the Scottish Intercollegiate Guidelines Network [29]. Nevertheless, it would be beneficial to continue research for specific cancer forms to build on this evidence further, especially bowel cancer.

#### 3.6.5. Subgroups of UPFs Including RTECs and Health Outcomes

As shown in Appendix B.6, nine studies explored RTECs/breakfast cereal consumption in the context of UPFs in relation to diet and health outcomes. Four publications were SRs or MAs [73,77,78,80]. In these, breakfast cereal consumption was associated with lower mortality risk (RR 0.85) [80], reductions in total cholesterol and LDL-c [77], and lower T2D risk (HR 0.78) [73]. Colds cereals were inversely associated with CVDs (HR 0.92), CHDs (HR 0.90) and stroke risk (HR 0.93) [78].

Five observational studies were also identified [24,81,82,83,84]. It should be noted that, in most of these publications, RTECs were grouped with other foods such as breads and biscuits, highlighting a need for well-defined sub-categories in future studies. Interestingly, an analysis of data from the European Prospective Investigation into Cancer study (EPIC) [81] identified that breakfast cereals (also grouped with breads and biscuits) were associated with a reduced risk of type 2 diabetes (HR 0.65). Research by Cordova et al. (2023) [24] also using EPIC data concluded that ultra-processed breads and cereals were not associated with cardiometabolic diseases (HR 0.97). In one cross-sectional study, breakfast cereal intake was inversely associated with an ultra-processed dietary profile [84].

Regarding gastrointestinal health, the effects were less clear cut. Ultra-processed breads and breakfast foods (which included cold breakfast cereals, breakfast bars, muffins and different bread varieties) were associated with high-risk polyps (HR 1.13) [82] and Crohn’s disease (HR 1.18) [83].

Overall the quality of the available studies investigating RTECs as ‘ultra-processed’ foods were classed as Grade A. Whilst RTECs may be categorised as UPFs according the NOVA food classification system, they have been associated with reduced mortality [80] and reduced risk of T2D, CVDs, CHDs and stroke [73,78] when analysed alongside other food categories.

## 4. Discussion

Breakfast has been viewed historically as the most important meal of the day and cereals have always had an important role to play as part of breakfast traditions [85]. For thousands of years, cereals have been processed to convert raw grains into safe, palatable and nutritious foods [13].

In collating high-quality evidence, this review contributes to the evidence base by taking a broadbrush approach to the role of RTECs in diet, health and NCD prevention. Overall, there is an established body of evidence from surveys, SR/MAs and PCSs that RTEC consumption can contribute positively to nutrient intakes, particularly fibre and whole grains. Any concerns raised in studies mostly related to children’s sugar intakes derived from RTECs. Thus, policy messaging to opt for whole-grain, high-fibre and lower-sugar cereals would help to address this. More studies are needed to quantify intakes of free sugars from different food sources, as a percentage of energy intake, so that these can be compared against national and WHO guidelines [40]. At present, some dietary surveys report free sugar (the percentage contribution of total sugars and free sugars to daily total intakes ranged from 2 to 11% for both; Table 3), whilst others reported added or total sugars, which makes cross-comparisons challenging. RTECs also provide a range of micronutrients such as vitamin A, B2, B6, folate, B12, vitamin D, calcium, magnesium, potassium, iron and zinc [59,60]. The addition of cow’s milk may also provide nutrients such as calcium, zinc, iodine and vitamins A, B2, B12 and D [86]. Adding fortificants appears to yield benefits, especially for at-risk groups such as women of childbearing age or young people where physiological demands widen gaps between nutrient intakes and recommendations [41].

Turning to health and NCD prevention, RTEC consumption appears to be most strongly related to reduced CVD risk, particularly when whole grain varieties are consumed [74,75,76,78]. RTEC consumption also appears to prevent T2D risk [25,72,73] and contribute to improvements in body weight and composition, amongst both children/young people and adults (Grade B) [26,69,70,71]. For all-cause cancer risk, there was one particularly strong MA, but this focused prominently on whole grain consumption, which included breakfast cereals, rather than RTECs specifically [76]. Nevertheless, a 90 g/day increase in whole grain intake which included breakfast cereals was associated with reduced total cancer risk, with reductions in risk observed in an intake of 210–225 g/day (equivalent to seven or seven and a half servings of whole grains daily) (Grade A) [76]. For food groups, breakfast/diet quality and blood lipids, there is a need for more well-designed studies with large sample sizes which control for confounders and separate RTECs from other foods groups, such as bread (Grade D). In addition, given that RTECs are typically consumed with milk, studies need to clarify whether the nutrients from milk have been accounted for [87]. With the increasing push towards plant-based diets, future studies should also consider the impact of consuming plant-based milks alongside RTECs.

The perception that RTECs are heavily processed and harmful to health requires careful examination and should be placed in a more rational context. Indeed, in several publications, RTECs were classified as ultra-processed, yet also associated with beneficial health outcomes including reduced mortality [80] and risk of T2D, CVDs, CHDs and stroke [73,78]. The lack of consistency between processing level and health effects for several categories of foods has led authors to demand that the classification of UPFs should be nuanced by adding a nutritional component [88,89]. An evaluation of 106 sets of dietary guidelines found that 99% encompassed ‘nutrient-based’ messaging, i.e., promoting beneficial nutrients such as vitamins or dissuading negative nutrients such as sugar, salt and fat. In contrast, ‘food-processing’ messages were only evident in 45% of ‘eat less’ and 5% ‘eat more’ guidelines [89]. As demonstrated by modelling research, optimised RTEC fortification has a key role to play in reducing the proportion of people below recommended intakes or above the UL [44]. It is almost impossible in modern economies to avoid the consumption of UPFs given the realities of food safety issues, time, cost, convenience, taste, acceptance and the increased engagement of women (traditionally given the role of food purchasing and preparation) in the workplace [88]. Therefore, categorisation and even demonisation of foods based simply on processing is at odds with current dietary recommendations in many countries, as well as consumer expectations of the types of diets that fit with their modern lifestyles and the need to feed 9.8 billion people by 2050 (projected to reach 11.2 billion by 2100) [90].

A limitation of the current review is that it was not possible to use one large centre database for the search. Two databases were used, given the large volume and breadth of studies that were extrapolated, though it should be recognised that this may have resulted in the omission of some publications. One strength was the detailed grading of studies for quality and the focus on SRMA and RCTs, which are at the top of the hierarchy of evidence. There was a general lack of consensus in study definitions and categorisation of breakfast cereals/RTECs. Mostly these were analysed in isolation, but, on some occasions, they were clustered with other foods such as breads. This can make it difficult to extrapolate data specifically related to RTECs. In observational studies, confounding by other dietary, lifestyle and environmental factors can influence study findings on disease risk and are not necessarily indicative of cause-and-effect relationships. This is why observational studies need to be augmented with RCTs and mechanistic studies.

In terms of future research directions, there is scope to research in detail the potential health effects and nutritional implications of consuming sub-categories of RTECs with varying nutritional profiles, e.g., brans, puffs, flakes, plain hot cereal and flavoured hot cereal [91], potentially comparing and contrasting these. Data could also be modelled and stratified by breakfast type or pattern. It would also be useful for future surveys and studies to put cereals into context with other sugar sources. Clear and uniform RTEC categorisation systems, e.g., without including other food groups such as cereals bars would also be beneficial. Finally, the use of dietary recall methods within studies can be subject to under- and over-reporting and should be considered. Nutritional biomarkers could also be used to validate dietary intake data. More studies focusing on markers of nutritional status alongside habitual intakes would also be beneficial.

## 5. Potential Mechanisms of Action

Several underpinning mechanisms could explain inter-relationships between RTECs, NCD prevention and markers of health. Firstly, in terms of constituents, RTECs provide dietary fibre which can vary from 10 to 15% [26]. They can also contain whole grains, dried fruit and soluble fibre, which may range from 20% (wheat) to 50% (oats) [26]. Beta-glucans from barley and/or oats can also be incorporated and found in RTECs [92]. The fibre profiles of cereal are fundamental to health and wellbeing [93]. This is of great significance, given that there are large gaps (about 10 g/day) in dietary fibre intakes versus recommendations in Westernised countries [40,51].

A substantial weight of evidence appears to relate to whole-grain breakfast cereals. From a mechanistic stance, this could be due to their resistant starch, fibre, polyphenols, antioxidant profiles, ability to bind carcinogens and modulate glycaemic response, and support optimal gut microbiota profiles [94,95].

It is well recognised that RTECs provide folic acid [96]. In turn, folic acid plays a role in regulating cardiovascular health by lowering homocysteine levels which, when elevated, increase CVD risk [97]. Dietary fibre [98] and cereal beta-glucans [99] have also been identified as possible factors helping to ameliorate CVD risk. Williams et al. (2014) reported that “regular consumption of oat-, barley- or psyllium-based breakfast cereals can help lower total and LDL cholesterol concentrations”, and rated this as grade A in terms of evidence [25].

Whole grains have also been associated with weight loss in adults via the fermentation of non-digestible carbohydrates (which can induce satiety signals), modulation of intestinal flora and reduction in glycaemic index [100]. Cereal beta-glucans may reduce body weight and adiposity by influencing satiety, altering gastric emptying time, gut hormones, gut microbiota and SCFAs which, in turn, can affect energy regulation and appetite [101].

Regarding glucose levels and prevention of T2D dietary, fibre and other bioactive compounds found in whole grains can help regulate glycemia, insulinemia, the gut microbiome and by-products of the gut microbiome [102]. In relation to cancer risk, whole grain consumption has been inversely linked to GI tumour risk, which may be attributed to its fibre and polyphenol profile [103], and the binding of carcinogens in faecal matter [94].

Finally, it should be considered that RTEC intake may simply be a marker of other lifestyle habits associated with a reduced risk of chronic disease, including physical activity, high fruit and vegetable intake, or reduced saturated fat intake [64,104].

## 6. Recommendations on Potential Policy Messages

There has been a growing body of high-quality evidence syntheses for RTECs in relation to various aspects of diet and health. Given the growing concerns about scientific interpretations from the NOVA classification of ultra-processed foods, government bodies and organisations should take a methodical and objective approach when translating this into policy messages [105,106,107,108]. So far, looking at the evidence-base and translating this into a public health perspective, we can draw the following conclusions.

*RTECs are a valuable provider of whole grains*—It is important to consider whole grains as a food group (similar to fruits and vegetables), whilst fibre is a nutrient and can be likened to protein, vitamins and minerals. Studies have shown that RTECs can be a valuable provider of whole grains. This, in turn, has been linked to beneficial health outcomes, such as improvements in body weight and reduced risk of T2D, CHDs and total cancer [76,100,109,110]. There is a need to enhance consumer awareness regarding how to identify whole-grain foods, the recommended intakes of whole grains, and the long-term health benefits associated with their consumption [111].

*Macronutrient profiles need to be communicated in context*—Overall, in the present review (Table 1), evidence from Western dietary surveys shows that RTECs contribute 2–10% of daily energy intake, just 1–4% of daily fat and saturated fat and, amongst consumers, 16–23% dietary fibre. Regarding total sugars, RTECs supplied around 8–11% of daily intakes in children and young people [32,36,37]. In adults, this was 8–10% [32,36], or 16% added sugars, as derived from US data [38]. Overall, this indicates that RTECs are low in fat, a good provider of fibre and can provide some free and total sugars. Dietary surveys rarely report on different types of mono- and disaccharides, so there are little data available on the contribution of RTECs to fructose intake, for example. Some earlier research found that adolescent RTEC consumers had higher intakes of glucose and fructose than bread consumers (3.1 g at breakfast versus 2.1 g), although fruit intake was also higher in this group, which may provide one explanation behind this [112]. Eating RTECs as part of a healthy and balanced diet, opting for whole-grain or lower-sugar varieties and avoiding adding table sugar, would be prudent advice and help to keep energy intakes and total sugar intakes in alignment with health policies.

*Certain population groups could benefit from the nutrient density provided by fortified RTECs*—We know from large dietary analyses that certain sub-groups are at risk of nutrient shortfalls, particularly when dietary requirements rise due to the physiological demands of ageing, pregnancy, lactation or growth. For example, a US NHANES analysis [113] of pregnant women (n = 1003, 2001–2014 dataset) showed that even with supplement use, at least 10% had habitual total usual intakes less than the estimated average requirement for vitamin A, B6, folate, C, D, E, calcium, magnesium, zinc and iron.

In the UK, even with supplements, we know that mean vitamin D intakes are only around 29–40% of the Reference Nutrient Intake for children [51]. Given these gaps and the micronutrient contributions that RTECs can provide [60], there is scope to integrate these within health and education policies. Eating RTECs is an easy and cost-effective way to improving diet quality, nutrient intakes and the nutrient density of diets [1,5,37,53,54,58,59,60].

*RTEC consumption may help to address dietary shortfalls in vitamin B12 and iron*—Shortfalls in vitamin B12 (cobalamin) are common globally in sub-populations with low consumptions of animal-foods, such as people avoiding animal foods for ethical, welfare or cost reasons [114]. Around 1.8 billion people have anaemia globally, with 60% cases attributed to iron deficiency, which can impact cognitive and physical development in children, as well as work productivity, and wellbeing [115]. For UK girls aged 11–18 years, 56% have iron intakes below the Reference Nutrient Intake, and 89% of women of childbearing age (16–49 years) have below-optimal red blood cell folate levels, which elevates the risk of neural tube defects [51]. As shown in the present review, RTECs may help to bridge some dietary gaps, providing around 39% of daily vitamin B12 and 51% iron intake in children and young people [37], and 38% vitamin B12 and 49% iron intake in adults [38].

*RTEC consumption may help to address dietary shortfalls in fibre*—Fibre-deficient diets have been reported globally [116]. Using the UK as an example, all age groups have mean fibre intakes that are below recommendations [51]. Mean daily intakes (19.7 g/day) in adults (19–64 years) are ≈10 g lower than benchmark dietary recommendations set at 30 g/day [40,51]. As identified in the present review, the consumption of RTECs can be associated with healthier dietary patterns, including improved whole grain and dietary fibre intakes [1,25,45,61]. In turn, dietary fibre intake is associated with lower risks for early mortality, metabolic disease, GI conditions (including constipation), colorectal carcinoma risk and gut dysbiosis [93].

*RTEC consumption may be associated with key aspects of health*—Guided by the strength of the evidence and quality of the studies available, breakfast/cold cereals were inversely associated with CVD risk [74,78], with the consumption of whole-grain breakfast cereals, in particular, being associated with reduced risk [75,76]. RTEC consumption and improvements in blood lipids levels were also observed in several studies [1,25,77]. For diabetes, there is promising evidence that RTEC consumption is associated with lower T2D risk [25,72,73]. RTEC consumption has been associated with reduced body weight, overweight/obesity and lower BMI in children, teens and adults [69,70], particularly whole grain varieties [26]. One good-quality MA [76] concluded that total whole grain consumption which included breakfast cereal may lower all-cause cancer risk (per 90 g daily whole grain increase). Now, there is a need for more research investigating RTEC consumption in relation to site-specific cancer forms, e.g., gastrointestinal carcinoma.

*Ultra-processing messaging in the context of RTECs can be confusing*—Findings from the present review show that there are growing concerns about the contradictions between nutrient- and processing-based dietary advice [89,106]. Therefore, as FBDGs evolve and begin to include reference to UPFs, it is important that nutrient-based messaging is not diluted or confused [89]. It has also been reported that universal definitions and classification systems of UPFs need updating to improve consumer understanding [117,118]. Erroneous messaging advising the exclusion of all UPFs—which include several useful and convenient foods—could have unintended consequences, such as reduced nutrient intakes in vulnerable population groups, such as the elderly or women of childbearing age [41]. Foods that are processed are not necessarily always less healthy or less sustainable which can be confusing for consumers and health professionals [119].

*Integration into policies*—It is important that public health nutrition policies continue to reflect the evidence base on nutritional composition and avoid being deflected into food processing categories. Straightforward nutritional messaging has long been applied and understood, backed by mechanistic studies [89]. Subsequently, reinforcing this approach, i.e., that RTECs are generally low in fat/saturated fat, provide dietary fibre and an array of vitamins and minerals including vitamin B12, folic acid and iron, could be a useful way forward. Sensible advice such as selecting lower-sugar varieties and avoid adding table sugar could be disseminated. Communications about processing levels are likely to add a layer of complexity not backed by mechanistic work or RCTs. In essence, such messaging should be treated with caution until the simplistic NOVA classification is properly evaluated.

## 7. Conclusions

RTECs are a low-fat, fibre dense food with the potential to benefit human health when consumed within a healthy, balanced diet within moderation. In particular, there is established evidence from survey and meta-analysis/systematic review publications that RTECs make significant contributions to nutrient intakes, particularly for certain population sub-groups such as children, young people and women of childbearing age, who have higher nutritional requirements. A strong body of evidence shows that RTEC consumption, particularly whole grain varieties, is associated with lower chronic disease risk and NCD prevention (CVDs and mortality and total cancer risk). A large body of observational evidence also associates RTEC consumption with reduced T2D risk and better weight management. Subsequently, despite being regarded as a processed food, RTECs do not appear to have a detrimental impact on health. Overall, the evidence indicates that incorporating higher-fibre, lower-sugar RTECs as part of the daily diet appear to benefit the nutritional density of diets and are an important dietary strategy that could help to prevent certain NCDs.

## Figures and Tables

**Table 1:** Levels of evidence using updated pyramid proposal and levels of scientific evidence according to SIGN.

Level of Evidence	Descriptor
Ma1++ Ma1+ Ma1−	Meta-analyses of high quality with a very low risk of bias. Meta-analyses well-conducted with a low risk of bias. Meta-analyses with a high risk of bias.
Rs1++ Rs1+ Rs1−	Systematic review of high quality with a very low risk of bias. Systematic review well-conducted with a low risk of bias. Systematic review with a high risk of bias.
RCT1++ RCT1+ RCT1−	RCTs of high quality with a very low risk of bias. RCTs well-conducted with a low risk of bias. RCTs with a high risk of bias.
Coh2++	Cohort studies with a very low risk of confounding or bias and a high probability that the relationship is causal.
Coh2+	Cohort studies with a low risk of confounding or bias and a moderate probability that the relationship is causal.
Coh2−	Cohort studies with a high risk of confounding or bias and a significant risk that the relationship is not causal.
CaCo2++	Cohort studies with a very low risk of confounding or bias and a high probability that the relationship is causal.
CaCo2+	Cohort studies with a low risk of confounding or bias and a moderate probability that the relationship is causal.
CaCo2−	Cohort studies with a high risk of confounding or bias and a significant risk that the relationship is not causal.

Source: Adapted from the work of Arieta-Miranda (2022) [29].

**Table 2:** Grades of recommendation using updated pyramid proposal and levels of scientific evidence according to SIGN.

The levels of scientific evidence categorised as “1++” and “1+” generate a “*type A*” recommendation degree. *Grade A*—A At least one meta-analysis, systematic review, or clinical study rated as 1++ and directly applicable to the target population, or a body of evidence consisting principally of studies rated as 1+, directly applicable to the target population, and demonstrating consistency of results.
The level of scientific evidence categorised as “2++” generates a “*type B*” recommendation degree. *Grade B*—A body of evidence including studies rated as 2++, directly applicable to the target population and demonstrating overall consistency of results, or extrapolated evidence from studies rated as 1++ or 1+.
The level of scientific evidence categorised as “2+” generates a “*type C*” recommendation degree. *Grade C*—A body of evidence including studies rated as 2+, directly applicable to the target population and demonstrating overall consistency of results, or extrapolated evidence from studies rated as 2++.
The levels of scientific evidence categorised as “3” and “4” generate a “*type D*” recommendation degree. *Grade D*—Evidence level 3 or 4 or extrapolated evidence from studies rated as 2+. Recommended best practice based on the clinical experience of a guideline development group.
The levels of scientific evidence categorised as “1−” and “2−” are considered “not recommended” due to high bias risk.

Source: Adapted from the work of Arieta-Miranda (2022) [29].

**Table 3:** Macronutrient intakes provided from breakfast cereals (% contribution to mean daily intake).

					Energy	Carbohydrate	Protein	Fat	Saturated Fat	Sugar	Fibre
Reference and Country	Age, y	Cereal	Analysis	% Consumers Only	%	%	%	%	%	%	%
NDNS RPS (2016/17–2018/19) years 9–11, 2020 UK [34]	4–10	High-fibre breakfast cereals	C+NC	NR	3	5	3	1	1	3 FS	7
NDNS RPS (2016/17–2018/19) years 9–11, 2020 UK [34]	4–10	Other breakfast cereals	C+NC	NR	2	4	1	1	1	4 FS	2
NDNS RPS (2016/17–2018/19) years 9–11, 2020 UK [34]	11–18	High-fibre breakfast cereals	C+NC	NR	2	3	2	1	1	3 FS	5
NDNS RPS (2016/17–2018/19) years 9–11, 2020 UK [34]	11–18	Other breakfast cereals	C+NC	NR	2	4	1	1	1	4 FS	2
NDNS RPS (2016/17–2018/19) years 9–11, 2020 UK [34]	19–64	High-fibre breakfast cereals	C+NC	NR	3	5	3	2	2	2 FS	6
NDNS RPS (2016/17–2018/19) years 9–11, 2020 UK [34]	19–64	Other breakfast cereals	C+NC	NR	2	2	0	0	0	2 FS	1
IUNA NCFS II (2017–18), Ireland [31]	5–12	Breakfast cereals	C+NC	85% RTEC 59% HFC 54% LFC 28% porridge/hot oat cereals (made up)	9	12	6	3	3	7 TS 11 FS	15
IUNA NTFS II (2019–2020), 2021 Ireland [33]	13–18	Breakfast cereals	C+NC	67% RTEC 45% HFC 37% LFC 17% porridge/hot oats	7	10	5	NR	NR	6 TS 11 FS	12
IUNA NANS II (2021–2022), 2024 Ireland [35]	19–64	Breakfast cereals	C+NC	39% RTEC 29% HFC 12% LFC 30% porridge/hot oats	5	8	4	NR	3	4 TS	8
IUNA NANS II (2021–2022), 2024 Ireland [35]	≥65	Breakfast cereals	C+NC	44% RTEC 33% HFC 17% LFC 47% porridge/hot oats	7	10	6	NR	4	5 TS	12
INCA 3 (2017), France [30]	1–10 11–17	Breakfast cereals and bars	C+NC	35% B (1–10 y) 37% G (1–10 y) 46% M (11–17 y) 50%F (11–17 y)	2 1	3 5	1 2	1 1	1 1	2 TS 4 TS	3 4
INCA 3 (2017), France [30]	18–79	Breakfast cereals and bars	C+NC	17%M 13%F	1	2	1	1	1	1 TS	1
CCHS 2015, Vatanparast et al., 2019, Canada [32]	2–12	RTECs	C	38% RTEC	8	11	4	3	2	8 TS	16
CCHS 2015, Vatanparast et al., 2019, Canada [32]	13–18	RTECs	C	29% RTEC	9	14	5	4	3	11 TS	19
CCHS 2015, Vatanparast et al., 2019, Canada [32]	≥19	RTECs	C	19% RTEC	9	15	6	3	3	10 TS	23
CCHS 2015 Sanders et al., 2024, Canada [36]	2–18	RTECs	C	36% RTEC LI 35% RTEC MI 36% RTEC HI	≈6 ≈7 ≈7	≈11 ≈12 ≈12	≈3 ≈3 ≈4	≈2 ≈2 ≈3	≈2 ≈1 ≈3	≈6 TS ≈7 TS ≈6 TS	≈14 ≈16 ≈17
CCHS 2015 Sanders et al., 2024, Canada [36]	19+	RTECs	C	22% RTEC LI 23% RTEC MI 21% RTEC HI	≈8 ≈8 ≈9	≈12 ≈12 ≈15	≈5 ≈4 ≈5	≈2 ≈3 ≈3	≈1 ≈3 ≈3	≈7 TS ≈8 TS ≈9 TS	≈22 ≈22 ≈23
NHANES 2015–16 Smith et al., 2019, USA [37]	0.5–17	RTECs	C	36% RTEC	≈10	≈14	≈4	≈3		≈18 AS ≈11 TS	≈18
NHANES 2015–16 Smith et al., 2019, USA [37]	0.5–17	RTECs	C+NC	--	≈3	≈6	≈2	≈1		≈6 AS ≈4 TS	≈7
NHANES 2015–16 Zhu et al., 2019, USA [38]	18+	RTECs	C	19% RTEC	10	16	6	3	--	16 AS	22
NHANES 2015–16 Zhu et al., 2019, USA [38]	18+	RTECs	C+NC	--	2	3	1	1	--	3 AS	5

Note: Data presented for males and females collectively. Key: AS, added sugar (defined as sugars that are added to foods and drinks during processing or preparation and excluding naturally occurring sugars in fruit, vegetables, dairy or juiced varieties [39]); FS, free sugars (defined as those added to food or those naturally present in honey, syrups and unsweetened fruit juices, but exclude lactose in milk and milk products [40]); TS, total sugars (defined as all mono- and disaccharides, regardless of source [39]). C, consumers; CCHS, Canadian Community Health Survey; F, female; HFC, high-fibre cereals (≥6 g/100 g); HI, high income; INCA, l’Institut national du cancer; IUNA, Irish Universities Nutrition Alliance; LFC, low-fibre cereals (<6 g/100 g); LI, low income; M, male; MI, middle income; NANS, National Adult Nutrition Survey; NC, non-consumers; NDNS; National Diet and Nutrition Survey; NHANES, National Health and Nutrition Examination Survey; NR, not reported; NTFS, National Teens’ Food Survey; RPS, Rolling Programme Survey; RTEC, ready-to-eat cereal; USA, United States of America; y, years.

**Table 4:** Micronutrient intakes provided from breakfast cereals (% contribution to mean daily intake).

Reference and country	Age, y	Cereal	Analysis	% Consumers Only	Vit A (μg)	Vit B_1_ (mg)	Vit B_2_ (mg)	Vit B_3_ (mg)	Vit B_5_ (mg)	Vit B_6_ (mg)	Folate (µg)	Vit B_12_ (µg)	Vit D (µg)	Ca (mg)	Mg (mg)	K (mg)	Fe (mg)	Zn (mg)
NDNS RPS (2016/17–2018/19) years 9–11, 2020, UK [34]	4–10	High-fibre breakfast cereals	C+NC	NR	1	NR	9	NR	NR	NR	9	NR	6	4	5	3	12	4
NDNS RPS (2016/17–2018/19) years 9–11, 2020, UK [34]	4–10	Other breakfast cereals	C+NC	NR	NR	NR	7	NR	NR	NR	9	NR	15	1	2	1	9	2
NDNS RPS (2016/17–2018/19) years 9–11, 2020, UK [34]	11–18	High-fibre breakfast cereals	C+NC	NR	0	NR	7	NR	NR	NR	6	NR	5	3	4	2	8	3
NDNS RPS (2016/17–2018/19) years 9–11, 2020, UK [34]	11–18	Other breakfast cereals	C+NC	NR	NR	NR	7	NR	NR	NR	8	NR	13	1	2	1	8	1
NDNS RPS (2016/17–2018/19) years 9–11, 2020, UK [34]	19–64	High-fibre breakfast cereals	C+NC	NR	1	NR	6	NR	NR	NR	5 *	NR	3	3	5	3	8	4
NDNS RPS (2016/17–2018/19) years 9–11, 2020, UK [34]	19–64	Other breakfast cereals	C+NC	NR	NR	NR	3	NR	NR	NR	3	NR	6	0	1	0	3	1
IUNA NCFS II (2017–18), Ireland [31]	5–12	Breakfast cereals	C+NC	85% RTEC 59% HFC 54% LFC 28% porridge/hot oat cereals (made up)	2	18	22	15	-	18	24	12	23	8	11	5	28	9
IUNA NTFS II (2019–2020), 2021, Ireland [33]	13–18	Breakfast cereals	C+NC	67% RTEC 45% HFC 37% LFC 17% porridge/hot oats	8	NR	18	NR	NR	NR	25	NR	19	5	NR	NR	22	NR
IUNA NANS II (2021–2022), 2024, Ireland [35]	19–64	Breakfast cereals	C+NC	39% RTEC 29% HFC 12% LFC 30% porridge/hot oats	NR	NR	9	NR	NR	6	9	6	6	6	NR	NR	10	6
IUNA NANS II (2021–2022), 2024, Ireland [35]	≥65	Breakfast cereals	C+NC	44% RTEC 33% HFC 17% LFC 47% porridge/hot oats	NR	NR	11	NR	NR	9	12	8	7	7	NR	NR	14	8
INCA 3 (2017), France [30]	1–10 11–17	Breakfast cereals and bars	C+NC	35% B (0–10 y) 37% G (0–10 y) 46% M (11–17 y) 50%F (11–17 y)	5 8	7 11	6 10	9 11	8 12	8 11	5 8	4 6	3 6	2 4	2 3	1 2	7 11	1 2
INCA 3 (2017), France [30]	18–74	Breakfast cereals and bars	C+NC	17%M 13%F	1	3	3	3	4	3	2	1	1	1	1	1	3	1
CCHS 2015, Vatanparast et al., 2019, Canada [32]	2–12	RTECs	C	38% RTEC	<0.01	23	4	9	NR	17	14 DFE	0	6	6	9	4	32	9
CCHS 2015, Vatanparast et al., 2019, Canada [32]	13–18	RTECs	C	29% RTEC	<0.01	30	8	11	NR	20	30 FA	0	9	6	11	5	35	10
CCHS 2015, Vatanparast et al., 2019, Canada [32]	≥19	RTECs	C	19% RTEC	<0.01	29	6	11	NR	17	16 DFE	0.01	5	6	12	6	32	12
CCHS Sanders et al., 2024, Canada [36]	2–18	RTECs	C	36% RTEC LI 35% RTEC MI 36% RTEC HI	NR NR NR	≈28 ≈27 ≈26	≈7 ≈6 ≈4	≈8 ≈8 ≈8	NR NR NR	≈15 ≈16 ≈15	13 DFE 12 DFE 12 DFE	NR NR NR	≈7 ** ≈7 ** ≈7 **	≈4 ≈4 ≈4	≈8 ≈9 ≈10	≈2 ≈3 ≈4	≈32 ≈32 ≈32	≈8 ≈9 ≈9
CCHS Sanders et al., 2024, Canada [36]	19+	RTECs	C	22% RTEC LI 23% RTEC MI 21% RTEC HI	NR NR NR	≈32 ≈29 ≈33	≈9 ≈5 ≈4	≈10 ≈9 ≈10	NR NR NR	≈16 ≈13 ≈15	15 DFE 12 DFE 13 DFE	NR NR NR	≈4 ** ≈3 ** ≈5 **	≈4 ≈4 ≈3	≈13 ≈12 ≈13	≈5 ≈5 ≈5	≈33 ≈31 ≈32	≈10 ≈10 ≈12
NHANES 2015–16 Smith et al., 2019, USA [37]	0.5–17	RTECs	C	36% RTEC	≈36	NR	≈27	≈35	--	≈43	34 FA	≈39	≈21	≈8	≈11	≈5	≈51	≈33
NHANES 2015–16 Smith et al., 2019, USA [37]	0.5–17	RTECs	C+NC	--	≈17	NR	≈12	≈15	--	≈20	≈12 DFE	≈19	≈11	≈3	≈4	≈2	≈24	≈14
NHANES 2015–16 Zhu et al., 2019, USA [38]	18+	RTECs	C	19% RTEC	27	33	24	30	--	41	≈12 DFE	38	21	6	13	7	49	30
NHANES 2015–16 Zhu et al., 2019, USA [38]	18+	RTECs	C+NC	--	8	8	6	6	--	10	≈54	11	6	1	3	1	14	7

Note: Data presented for males and females collectively. C, consumers; CCHS, Canadian Community Health Survey; F, female; DFE, dietary folate equivalents; F, female; FA, folic acid; HFC, high-fibre cereals (≥6 g/100 g); HI, high income; INCA, l’Institut national du cancer; IUNA, Irish Universities Nutrition Alliance; LFC, low-fibre cereals (<6 g/100 g); LI, low income; M, male; MI, middle income; NANS, National Adult Nutrition Survey; NC, non-consumers; NDNS; National Diet and Nutrition Survey; NHANES, National Health and Nutrition Examination Survey; NR, not reported; NTFS, National Teens’ Food Survey; RPS, Rolling Programme Survey; RTEC, ready-to-eat cereal; USA, United States of America; y, years. * Folate for women of childbearing age (16–49 years) 4% high-fibre breakfast cereals, 3% other breakfast cereals; ** Vitamin D_2_ an.

## Data Availability

Data or models were not deposited in an official repository. No new datasets were created.

## Abbreviations

The following abbreviations are used in this manuscript:

AS	Added Sugars
BMI	Body Mass Index
BQI	Breakfast Quality Index
BQS	Breakfast Quality Score
C	Consumers
CCHS	Canadian Community Health Survey
CHD	Coronary Heart Disease
CI	Confidence Interval
CS	Cross-Sectional
CVD	Cardiovascular Disease
DALYs	Disability-adjusted life-years
DFE	Dietary Folate Equivalents
DQI	Diet Quality Index
EAR	Estimated Average Requirement
EI	Energy Intake
EPIC	European Prospective Investigation into Cancer
FBDGs	Food-Based Dietary Guidelines
FS	Free Sugars
GBD	Global Burden of Disease
GI	Glycemic Index
HEI	Healthy Eating Index
HELENA	Healthy Lifestyle in Europe by Nutrition in Adolescence
HFC	High-Fibre Cereals
HI	High Income
HR	Hazard Ratio
IBRI	International Breakfast Research Initiative
INCA	l’Institut National du Cancer
IUNA	Irish Universities Nutrition Alliance
LDL-C	Low-Density Lipoprotein Cholesterol
LFC	Low-Fibre Cereals
LI	Low Income
MA	Meta-Analysis
MD	Mean Difference
MI	Middle Income
NANS	National Adult Nutrition Survey
NC	Non-Consumers
NCD	Noncommunicable diseases
NCFS	National Children’s Food Survey
NDNS	National Health and Nutrition Examination Survey
NTDs	Neural Tube Defects
NTFS	National Teens’ Food Survey
PCS	Prospective Cohort Studies
PIR	Poverty-to-Income Ratio
PMID	PubMed ID
PRISMA	Preferred Reporting Items for Systematic Reviews and Meta-Analyses
RCTS	Randomised Controlled Trials
RNI	Reference Nutrient Intake
RPS	Rolling Programme Survey
RR	Relative Risk
RTEC	Ready-To-Eat Cereal
SCFAs	Short-Chain Fatty Acids
SDS	Slowly Digestible Starch
SIGN	Scottish Intercollegiate Guidelines Network
SMD	Standard Mean Difference
SNDA-II	School Nutrition Dietary Assessment Study
SR	Systematic Review(s)
SR/MA	Systematic Review/Meta-Analyses
TS	Total Sugars
T2D	Type 2 Diabetes
UK NDNS	United Kingdom National Diet and Nutrition Survey
UL	Upper Limit
UPF	Ultra-Processed Food
US NHANES	United States National Health and Nutrition Examination Survey
WIC	Special Supplemental Nutrition Programme for Women, Infants, and Children
y/yrs	Years

## Appendix A

### Applied Search Words and Terminologies

**Table:**

“ready-to-eat cereal” [tiab] OR “RTE cereal” [tiab] OR “RTEC” [tiab] OR “breakfast cereals” [tiab] AND “modelling” [tiab]
“ready-to-eat cereal” [tiab] OR “RTE cereal” [tiab] OR “RTEC” [tiab] OR “breakfast cereals” [tiab] AND “food groups” [tiab]
““ready-to-eat cereal” [tiab] OR “RTE cereal” [tiab] OR “RTEC” [tiab] OR “breakfast cereals” [tiab] AND “breakfast quality” OR “diet quality” [tiab]
“ready-to-eat cereal” [tiab] OR “RTE cereal” [tiab] OR “RTEC” [tiab] OR “breakfast cereals” [tiab] AND “nutrient intakes” [tiab] OR “nutritional adequacy” OR “micronutrient” [tiab]
“ready-to-eat cereal” [tiab] OR “RTE cereal” [tiab] OR “RTEC” [tiab] OR “breakfast cereals” [tiab] AND “body weight” [tiab]
“ready-to-eat cereal” [tiab] OR “RTE cereal” [tiab] OR “RTEC” [tiab] OR “breakfast cereals” [tiab] AND “glucose levels” [tiab]
“ready-to-eat cereal” [tiab] OR “RTE cereal” [tiab] OR “RTEC” [tiab] OR “breakfast cereals” [tiab] AND “type 2 diabetes” [tiab] OR “T2DM” [tiab]
“ready-to-eat cereal” [tiab] OR “RTE cereal” [tiab] OR “RTEC” [tiab] OR “breakfast cereals” [tiab] AND “cardiovascular disease” [tiab] OR “CVD” [tiab]
“ready-to-eat cereal” [tiab] OR “RTE cereal” [tiab] OR “RTEC” [tiab] OR “breakfast cereals” [tiab] AND “lipid*” [tiab]
“ready-to-eat cereal” [tiab] OR “RTE cereal” [tiab] OR “RTEC” [tiab] OR “breakfast cereals” [tiab] AND “cancer” [tiab]
“ready-to-eat cereal” [tiab] OR “RTE cereal” [tiab] OR “RTEC” [tiab] OR “breakfast cereals” [tiab] AND “ultra process*” [tiab]

Time restriction from 1 January 2004 to 16 September 2024.

## Appendix B

### Appendix B.1. Modelling Studies Focusing on RTECs/Breakfast Cereals

**Table:**

Author, Year, PMID	Country	Dataset	Model/Approach	Number of Participants	Main Focus	Results
Tucker et al. (2024) [46] NA	United States	NHANES 2011–2018	Modelling replaced RTEC at different levels (10, 25, 50, 100%) with a weighted composite of all breakfast intakes except RTEC.	n = 9292, 2–18 y	Added sugars, whole grain and nutrients	Replacing 100% of RTECs with other breakfast foods yielded minimal reductions in daily AS intakes and reduced intakes of whole grains, fibre, and micronutrients.
Estell et al. (2022) [41] PMID: 34668030	Australia	National Nutrition and Physical Activity Survey 2011–12	Dietary modelling examined the nutritional adequacy of sample diets including grain-based UPFs as aligned with Australian Dietary Guidelines and another containing replacements for grain-based UPF.	n = 12,153	Nutrient intakes	There was a significant decline (*p* < 0.05) in the modelled intake of key nutrients when grain-based UPFs were excluded, especially thiamine, folate and iodine, as non-grain substitutions were rarely fortified.
Papanikolaou and Fulgoni (2021) [42] PMID: 34552951	United States	NHANES 2009–2016	Estimated usual daily intake of shortfall nutrients in the current dietary pattern and when specific percentages (25, 50 and 100%) of fortified/enriched refined grain foods (inc. RTECs) were removed from the diet.	n = 11,169, 19–50 y n = 9641, 51–99 y	Nutrient adequacy	Removal of bread and RTECs increased the percentage of adults not meeting the EAR for magnesium, vitamin A, C and E.
Calame et al. (2020) [43] PMID: 32585847	United Kingdom	NDNS: 2008–2012	4 cohorts assessed. The impact of 4.2 μg vit. D fortification per 100 g of RTEC on vit. D intake and status mathematically modelled.	n = 803, 4–10 y n = 884, 11–18 y n = 1655, 19–64 y n = 428, 65 y+	Vit. D status	Fortification of breakfast cereals can contribute to improving overall vitamin D status. Amongst males aged 4–10, 11–18, 19–64 and 65+ years, there was a 1.04, 3.21, 2.61 and 6.98 nmol/L difference in 25(OH)D levels with compared to without vitamin D fortification. Amongst females aged 4–10, 11–18, 19–64 and 65+ years there was a 1.26, 2.68, 2.70 and 5.55 nmol/L difference in 25(OH)D levels with compared to without vitamin D fortification.
Smith et al. (2020) [44] PMID: 31957633	United States	NHANES 2013–2014	Used 3 modelling scenarios: (1) baseline fortification, (2) zero fortification and (3) optimised fortification.	n = 559 toddlers, 1–3 y n = 1540 children, 4–12 y n = 992 teens, 13–18 y n = 576 adults, ≥19 y	Nutrient intakes	Fortification of RTEC can be optimised to provide key nutrients and minimise the percentage of the population below the EAR and above the UL.
Rehm and Drewnowski (2017) [45] PMID: 28902145	United States	NHANES 2007–2010	Model 1 solid foods eaten at breakfast replaced with RTECs on a calorie-per-calorie basis. Model 2 replaced solid breakfast foods with RTECs and milk, on a calorie-per-calorie basis. Beverages and beverage additions were not replaced.	n = 18,112	Nutrient intakes	Model 1 diets were sig. higher in folic acid (+104.6%), whole grains (+84.6%), iron (+54.5%), fibre (+14.3%), vitamin D (+14.0%), sugar (+5.0%) and lower in solid fats (−10.9%) compared to observed diets. Model 2 diets were additionally higher in dairy (+15.8%), calcium (+11.3%) and potassium (+3.95%).

Key: assoc, associated; BMI, body mass index; DFE, dietary folate equivalents; EAR, estimated average requirement; esp., especially; inc., including; LDL, low-density lipoprotein; NDNS, National Diet and Nutrition Survey; NHANES, National Health and Nutrition Examination Survey; RTE, ready-to-eat; sig, significantly; UL, Tolerable Upper Intake Level; UPF, ultra-processed food; vit., vitamin; yrs. Years.

### Appendix B.2. Studies Investigating RTEC/Breakfast Cereal Consumption and Food Groups

**Table:**

Author, Year, PMID	Country	Study Design	Number of Studies (Number of Participants)	Results
Smith et al. (2022) [53] PMID: 35425801	United States	Cross-sectional, US nationally representative 2015–2016 and 2017–2018 NHANES	n = 5028, 2–18 y	For children, there were positive associations between RTEC consumption and whole grain and dairy, (*p* < 0.001) intake. For adults both RTEC and PIR were positively assoc. with whole grain, dairy, and fruit (*p* < 0.001).
Zhu et al. (2022) [5]	United States	Data from NHANES 2017–2018	n = 2259, 2–18 y n = 4776, 19+ y	Children and adults consuming RTECs for breakfast had higher intakes of whole grains, and total dairy compared to consumers of non-RTEC breakfast or no breakfast.
Smith et al. (2019) [37] PMID: 31443588	United States	Cross-sectional analysis of data from NHANES 2015–2016	n = 88 RTEC C, 0.5–2 y n = 236 RTEC NC, 0.5–2 y n = 688 RTEC C, >2 to 12 y n = 1169 RTEC NC, >2 to 12 y n = 242 RTEC C, 13–17 y n = 546 RTEC NC, 13–17 y	Children (0.5–17 y) who ate RTECs had a 29% higher total dairy intake (*p* < 0.0001) and 61% higher whole grain intake (*p* < 0.0001). RTEC eaters had lower intake of meat, poultry, and seafood (*p* = 0.0005), eggs (*p* = 0.0009), but there were no differences in the intake of nuts and seeds, soybean products, or legumes
Michels et al. (2015) [54] PMID: 25403942	Multi-centre European study	Multi-centre European HELENA study	n = 1215, 12.5–17.5 y	RTEC consumers had a more frequent intake of milk/yoghurt and fruit (*p* < 0.001). Among RTEC consumers there was a higher percentage of consumers of fruit (57 vs. 51%) and milk/yoghurt (81.2 vs. 56%) compared to non-consumers. RTEC consumers were 2.65 times more likely to fulfil the daily milk intake recommendation than RTEC non-consumers (*p* < 0.001).
Affenito et al. (2013) [55] PMID: 23253288	United States	Cross-sectional data from the third School Nutrition Dietary Assessment Study, 2004–2005	n = 2298, 5–18 y	Pupils eating RTECs at breakfast and participating in the School Breakfast Programme consumed significantly more whole grains (0.71 oz. equiv) than pupils eating a non-cereal breakfast (0.43 oz. equiv), indicating that the School Breakfast Programme had an important role to play in improving intakes of certain food groups.

Key: assoc. associated; C, consumers; HELENA, Healthy Lifestyle in Europe by Nutrition in Adolescence; NC, non-consumers; NHANES, National Health and Nutrition Examination Survey; PIR, poverty-to-income ratio; RTE, ready-to-eat; y, years.

### Appendix B.3. Studies Investigating RTEC/Breakfast Cereal Consumption and Breakfast/Diet Quality

**Table:**

Author, Year, PMID	Country	Study Design	Number of Studies (Number of Participants)	Breakfast/Diet Quality Indices	Results	Level of Evidence
Priebe and McMonagle (2016) [1] PMID: 27749919	NA	SR of RCT and prospective studies	8 prospective studies (n = 154,217)	NA	Consumption of RTECs was associated with a healthier dietary pattern. Frequent RTEC consumption (≥5 servings/week) compared to low or no RTEC consumption was consistently associated with a healthier dietary pattern in children and adults in most studies demonstrating a higher consumption of carbohydrates, dietary fibre and a reduction in total fat intake and cholesterol (only for children). Therefore, current dietary recommendations are more likely to be met by RTEC consumers.	Rs1−
Harris et al. (2011) [56] PMID: 21149436	United States	1-day randomly assigned experimental design on camp where children had a choice of high-sugar cereals, low-sugar cereals or fruit/juice/milk options.	n = 91 children 5–12 y	Inter-associations determined between high/low sugar cereal consumption and other foods eaten at breakfast.	Children in the low-sugar RTEC group were more likely to put fruit on their cereal (54% vs. 8%) and consume a greater portion of total calories from fresh fruit (20% vs. 13%) compared to those eating high-sugar cereals. Compared with serving low-sugar RTECs, high-sugar RTECs increased children’s total sugar consumption and reduced the overall nutritional quality of their breakfast.	RCT2-
Poinsot et al. (2024) [57] PMID: 39021597	France	Data from the French representative cross-sectional (INCA3) dietary survey.	n = 1448, 4–17 y n = 4015 breakfasts	BQS tested by correlations with nutritional indicators and comparison of nutrients and dietary components between tertiles of scores. The BQS ranges from 0 to 100, 100 means that breakfast complied with all of IBRI recommendations.	RTEC breakfasts had the highest BQS (73.5% for children and 73.1% for teenagers) and biscuits and viennoiseries scored the lowest (52% for children and 49.1% for teenagers). This could be attributed to their fibre and micronutrient profile, as well as the co-consumption with milk, which was highest in RTEC breakfasts	NA
Bellisle et al. (2018) [58] PMID: 30096946	France	Nationally representative cross-sectional study	n = 426, children n = 250, adolescents n = 1045 adults	Measured by tertiles of the Nutrient-Rich Food Index 9.3 in children and adolescents (up to 17 y) and adults (18 y+)	Breakfasts of the highest population tertiles contributed more fibre and many vitamins and minerals for the same amount of energy. The highest tertiles consumed more milk, RTE breakfast cereals (notably whole-grain cereals) and bread; the highest tertile of the adult population had more dairy products and fruit, but less “viennoiseries” than lower tertiles.	NA
Michels et al. (2015) [54] PMID: 25403942	Multi-centre European study	Multi-centre European HELENA study.	n = 1215, 12.5–17.5 y	Diet quality calculated from 24 h dietary recalls using the DQI for Adolescents with Meal index (DQI-AM), which assessed the compliance with the Flemish FBDGs. The DQI-AM comprising four pillars: dietary quality, dietary diversity, dietary equilibrium and a meal index	Regarding diet quality, equilibrium and meal index, RTEC non-consumers scored lower than the frequent and daily RTEC consumers. Daily RTEC consumers had a significantly higher DQI score than non-consumers (*p* = 0.003). Frequent RTEC consumers (eating these 2–4 times a week) also had a significantly higher DQI score than non-consumers (*p* = 0.016).	NA
Smith et al. (2022) [53] PMID: 35425801	United States	Cross-sectional, US nationally representative 2015–2016 and 2017–2018 NHANES data	n = 5028, 2–18 y n = 9813 19+ y	Diet quality was measured using the HEI-2015 measuring how aligned an individual’s daily dietary intake is with the recommendations of the 2015 Dietary Guidelines for Americans. The maximum HEI score is 100 (highest-quality diet) based on the sum of 13 sub-scores for the intake of total fruits, whole fruit, total vegetables, greens and beans, whole grains, dairy, total protein foods, seafood and plant proteins, fatty acids, refined grains, sodium, added sugar, and saturated fats.	RTEC consumption was significantly associated with higher diet quality for children and adults. For 2015 subcomponents, RTEC consumption, for children and adults, was associated with a higher score (more aligned with dietary guidance) for whole grains (*p* < 0.0001) total dairy (*p* < 0.0001), whole fruit (adults only, *p* < 0.0001), sodium (*p* ≤ 0.0001), refined grains (*p* ≤ 0.0006), and saturated fat (*p* ≤ 0.0003) and lower scores (less aligned with guidelines) for the total protein foods (*p* ≤ 0.0003), fatty acids (*p* ≤ 0.0001), and added sugar (children only, *p* = 0.0024) components.	NA
Smith et al. (2019) [37] PMID: 31443588	United States	Cross-sectional, US nationally representative 2015–2016 NHANES data	n = 2969	Dietary quality measured using the HEI-2015. As above (Smith et al., 2022) [53].	Children who ate RTECs had a better diet quality than those who did not, as reflected by the HEI-2015 total score (52.6 versus 47.7, *p* < 0.0001).	NA

Key: BQI, breakfast quality index; BQS, breakfast quality score; DQI, diet quality index; FBDGs, food-based dietary guidelines; FFQ, food frequency questionnaire; HEI, Healthy eating Index; HELENA, Healthy Lifestyle in Europe by Nutrition in Adolescence; inc. increased; IBRI, International Breakfast Research Initiative; NA, not applicable; NHANES, National Health and Nutrition Examination Survey; RCT, randomised controlled trial; RTE, ready-to-eat; Sig. significant/ly; SR, systematic review; US, United States.

### Appendix B.4. Studies Investigating RTEC/Breakfast Cereal Consumption and Nutrient Intakes/Status

**Table:**

Author, Year, PMID	Country	Study Design	Number of Studies (Number of Participants)	Outcomes	Results	Level of Evidence
Giménez-Legarre N et al. (2020) [59] PMID: 32824257	NA	SR and MA of breakfast characteristics in children and teens.	38 studies (n = 120,193)	Energy, macronutrients	Children who usually skip breakfast had a lower daily EI (Kcal) than children eat RTEC (MD, −7.00; 95% CI: −11.51, −2.49). Children who usually skip breakfast had a sig. lower carbohydrate intake than children who usually consume RTECs (MD, −9.28; 95% CI: −13.44, −5.12). RTEC consumers had a significantly higher fibre intake than breakfast skippers (MD, −6.67; 95% CI: −11.02, −2.32). Breakfast skippers consumed less protein than those eating RTECs for breakfast (MD, −3.03; 95% CI: −4.61, −1.45). Children skipping breakfast had a significantly higher fat intake than children eating RTECs (MD, 11.10; 95% CI: 7.15, 15.04).	Rs/Ma1++
Giménez-Legarre N et al. (2020) [60] PMID: 33092061	NA	SR and MA of breakfast characteristics in children and teens.	33 studies–systematic review (n = 85,023) 7 studies–meta-analysis (NR)	Micronutrient intakes	Several articles assessed the consumption of RTECs (n = 22, 66.6%) In children, RTEC consumers had significantly higher daily consumption of thiamine (vitamin B1) than children who usually skip breakfast (SMD, −16.378; 95% CI: −29.110, −3.647). RTEC consumers also had significantly higher daily intake of riboflavin (vitamin B2) than children who usually skip breakfast (SMD, −14.757; 95% CI: −20.247, −9.268). Children who usually consume RTECs had a significantly higher intake of vitamins A and C than those who usually skip breakfast (SMD, −10.407; 95% CI: −14.147, −6.667 and SMD, −4.127; 95% CI: −5.091, −3.162), respectively. Children who usually ate RTECs at breakfast had significantly higher daily consumption of calcium and iron than children who skipped breakfast (SMD, −12.650; 95% CI: −14.616, −10.685 and SMD, −19.534; 95% CI: −27.887, −11.181, respectively) Children eating RTECs had a significantly higher magnesium and potassium intake than breakfast skippers (SMD, −10.903; 95% CI: −18.078, −3.729) and (SMD, −6.972; 95% CI: −10.689, −3.254), respectively. No differences were observed for sodium.	Rs/Ma1++
Priebe MG and McMonagle (2016) [1] PMID: 27749919	NA	SR of RCT and prospective studies.	8 prospective studies (n = 154,217)	Micronutrient intakes	Those consuming RTECs frequently (≥5 times/week) had a lower risk of inadequate micronutrient intakes especially for vitamin A, calcium, folate, vitamin B 6, magnesium and zinc compared to non-consumers. When using datasets that assessed significance, in children/adolescents and adults, reductions in prevalence of inadequacy due to RTEC consumption were highest for: vitamin A (range: 7–21% and 5–37%, respectively), calcium (17–39% and 6–40%, respectively), folate (5–28% and 7–50%, respectively), magnesium (7–11% and 4–26%, respectively) and zinc (9% and 19–37%, respectively). In adults, high reductions were also seen for vitamin B 6 (7–55%) and C (6–21%).	Rs1−
Williams et al. (2014) [25] PMID: 25225349	NA	SR	11 intervention trials included (n = 730)	Vitamin and mineral intakes	Children, adolescents and adults who consume RTE breakfast cereals regularly have daily diets that are higher in percentage of energy (%E) from carbohydrate, total sugars, dietary fibre, vitamins A and D, thiamine, riboflavin, niacin, pyridoxine, folate, calcium, iron, magnesium, and zinc; no different in total energy intake, %E from protein, or sodium; and are lower in %E from fat. Children and teens who eat breakfast cereal regularly are less likely to have vitamin and mineral intakes below the recommended daily requirements, especially for calcium. Adults who eat breakfast cereal regularly are less likely to have vitamin and mineral intakes below the recommended daily requirements, especially for thiamine, riboflavin, niacin, folate, vitamin C, calcium, magnesium, iron, zinc, and fibre.	Rs1−
Kuriyan et al. (2017) [61] PMID: 28917231	India	Two-week randomised, controlled 2-arm trial. The intervention group received 30 g of low-fat RTECs (120 mL skim milk) and a serving of fruit/veg., replacing two meals/day for two weeks. The control group was provided with standard dietary guidelines for weight loss.	n = 101 overweight/obese females, 18–44 y	Nutrient intake profile	The low-fat RTEC intervention group had a sig. higher increases in dietary intakes of certain vitamins, fibre and sugar, and significantly higher reductions in total and polyunsaturated fats and sodium intakes, as compared to the control group (*p* ≤ 0.05).	RCT2+
Powers et al. (2016) [62] PMID: 27418034	UK	Twelve-week randomised, double-blind, placebo-controlled intervention trial. Receive 50 g fortified or unfortified cereal, with 150 mL semi-skimmed milk, daily, as a breakfast or as a supper.	n = 73, 16–19 y	Micronutrient intakes	Consumption of fortified RTECs increased vitamins B1, B2, niacin, B6, B12, folate and iron (*p* < 0.001) and of vitamin D (*p* = 0.007) compared to the unfortified cereal.	RCT2++
Albertson et al. (2009) [64] NA	US	Secondary analyses using data from the Dietary Intervention Study in Children, a randomised, controlled, multicentre, trial.	n = 660, 8–10 y	Nutrient intake	RTEC consumption was positively associated with all measures of nutrients for both sexes.	RCT1+
Ortega et al. (2006) [63] PMID: 17010231	Spain	Dietary intervention study. Randomly assigned to one of two slightly hypocaloric diets: diet V (inc. veg. consumption) or diet C (inc. a minimum of 3 serves of breakfast cereals or cereal bars per day).	n = 67 overweight/obese women of childbearing age, 20–35 y.	Serum folate concentrations	At the start of the study, 64·2% of all subjects had a folate intake of <67% of the recommended intake; this fell to 3% (7·14% of V subjects and 0% of C subjects) by week 6. 62·1% of all subjects had serum folate concentrations of ≥13·6 nmol/L (associated with a very low risk of NTDs) at the start of the study, while 87·0% (85·2% of V subjects and 88·9% of C subjects) had concentrations of ≥13·6 nmol/L at 6 weeks (*p* < 0·01). These results indicate that breakfast cereals may help with folate intake/status in women of childbearing age.	RCT2++
Smith et al. (2022) [53] PMID: 35425801	US	Cross-sectional, US nationally representative 2015–2016 and 2017–2018 NHANES	n = 5028, 2–18 y n = 9813, 19 y+	Vitamin and mineral intakes	There was a sig. interaction between PIR and RTECs for adults for iron, phosphorus, B vitamins, and dairy (*p* < 0.001) with PIR category being positively associated with the intake of nutrients. RTECs contributed to one quarter or more of daily intake, across all age and PIR groups, for several B vitamins, iron, zinc, and whole grains.	NA
Zhu et al. (2022) [68] NA	US	Cross-sectional, US 2013–2016 NHANES	n = 531, 1–5 y	Nutrient intakes	In total, 45% of women, infants and children ate RTECs. Consumption in children was linked to significantly higher intakes of calcium (18%), iron (75%), zinc (47%), vitamin A (36%) thiamine (39%), riboflavin (32%), niacin (41%), vitamin B6 (53%), folate (116%), vitamin B12 (57%), and vitamin D (28%) compared to non-consumers.	NA
Zhu et al. (2021) [66] NA	US	Data from NHANES 2017–2018	n = 2135, 2–17 y n = 3675, 18–64 y n = 1221, 65 y+	Nutrient intakes	RTEC consumption was associated with significantly higher intake of calcium, iron, zinc, magnesium, potassium, phosphorus, vitamin A, thiamine, riboflavin, niacin, vitamin B6, folate, vitamin B12, and vitamin D (all *p* < 0.05),	NA
Zhu et al. (2020) NA [67]	US	Cross-sectional US NHANES 2013–2014	n = 2553, 1–18 y n = 4901, 19 y+	25-hydroxyvitamin D status	Children and adults eating RTECs had significantly (*p* < 0.05) higher levels of serum 25-hydroxyitamin D than those not eating RTEC, and adults were less likely to have vitamin D deficiency.	NA
Lepicard et al. (2017) [65] PMID: 27714860	France	Data collected during a cross-sectional observational study.	n = 529 French children, 9–11 y	Nutrient density	The RTEC + milk breakfast pattern was the most nutrient dense–it was lower in total fat, saturated fatty acids, and cholesterol and was rich in fluids, vitamins B, vitamin C, calcium, and iron.	NA
Michels et al. (2015) [54] PMID: 25403942	Multi-centre European study	Multi-centre European HELENA study.	n = 1215, 12.5–17.5 y	Micronutrient intakes	RTEC consumers had a more favourable daily micronutrient intake (vitamin B2, B5, B7, D, calcium, phosphorus and potassium) compared to non-consumers.	NA
Affenito et al. (2013) [55] PMID: 23253288	US	Data from the third cross-sectional School Nutrition Dietary Assessment Study, 2004–2005.	n = 2298, 5–18 y	Macro- and micronutrient intake	Protein, total sugars and sodium were no different between the breakfast groups. Pupils participating in the School Breakfast Programme and eating RTECs at breakfast consumed significantly more vitamin A (728 vs. 572 µg RAE), iron (19 vs. 14 mg) and dietary fibre (16 vs. 14 g) compared with those who ate a non-cereal breakfast.	NA

Key: Assoc, associated; BMI, body mass index; CI, confidence interval; EI, energy intake; MA, meta-analysis; NA, not applicable; NTDs, neural tube defects; PIR, poverty-to-income ratio; RCT, randomised controlled trial; RTE, ready-to-eat; Sig. significant/ly; SMD, standard mean difference; SR, systematic review; y, years.

### Appendix B.5. Systematic Reviews and Meta-Analysis Studies Investigating RTEC/Breakfast Cereal Consumption and NCD Risk/Aspects of Health

**Table:**

Author, Year, PMID	Study Design	Number of Studies (Number of Participants)	Outcomes	Results	Level of Evidence
Body weight (6 publications)					
de la Hunty et al. (2013) [71] PMID: 23466487	SR and MA	11 cross-sectional (n = 30,019) 2 prospective (n = 3039) 1 intervention (n = 147)	Body weight of children and teens	Overweight prevalence and risk of overweight was lower in children and teens who ate breakfast cereals regularly compared to those eating them infrequently.	Rs/Ma1+
Sanders et al. (2023) [69] PMID: 37149263	SR of observational studies and controlled trials	14 RCTs (n = 1184) 14 observational studies (n = 298,233)	Adult body weight	RTEC consumers (≥4 servings/wk)) had a lower BMI, prevalence of overweight/obesity, less weight gain and less anthropometric evidence of abdominal adiposity compared with NC, or less frequent consumers. RCTs suggest that RTECs may be used as a meal or snack replacement as part of a hypocaloric diet. RTEC consumption was not associated with sig. less loss of body weight, or with weight gain, in any of the RCTs.	Rs1+
Sanders et al. (2023) [70] PMID: 36811587	SR of observational studies and controlled trials	5 RCTs (n = 541) 20 observational studies (n = 65,052)	Body weight and composition of children and teens	Overall, 14 out of 20 observational studies found that children and teens eating RTECs had a lower BMI, prevalence and odds of overweight/obesity and more favourable indicators of abdominal obesity than NC/less frequent consumers. Controlled trials were few–one reported a loss of 0.9 kg in overweight/obese children with RTEC consumption when accompanied by nutrition education.	Rs1+
Kosti et al. (2010) [26] PMID: 20819244	SR	12 studies children and adolescents (2 RCTs n = 2179, 8 cross-sectional n = 8272, 2 prospective n = 4758) 9 studies in adults (4 RCTs n = 564, 2 cross-sectional n = 20,670, 3 prospective n = 119,054))	Obesity/adiposity in children, adolescents and adults	RTEC consumption was associated with a desirable macronutrient profile for obesity prevention, reduced BMI and weight gain (mainly for whole-grain cereals) and could be a potential meal replacement/alternative snacking option in weight-loss programmes.	Rs1+
Priebe and McMonagle (2016) [1] PMID: 27749919	SR	9 RCTs (n = 551) 3 prospective studies (n = 19,166)	BMI/Body weight/weight gain	Two RCTs did not find RTEC related to changes in body weight. 7 RCTs examined the effect of low-DF vs. high-DF RTECs and/or wholemeal RTECs on postprandial satiety and five on subsequent energy intake. Three trials reported a sig. difference in satiety/appetite measures. The degree of hunger was lower after ingestion of high- versus low-DF RTECs. Negative inter-relationships between frequent RTEC consumption with body weight gain were found in 3 prospective studies.	Rs1−
Williams et al. (2014) [25] PMID: 25225349	SR	3 systematic reviews 16 intervention trials focusing on weight management (n = 861)	Weight management	Regular breakfast cereal consumption was associated with a lower body mass index and less risk of being overweight or obese (evidence grade B)	Rs1−
Glucose Levels and Type 2 Diabetes (4 publications)					
Aune et al. (2013) [72] PMID: 24158434	MA of cohort studies	Three cohort studies focused on whole-grain breakfast cereals (n = 137,142)	Type 2 diabetes	Inverse associations observed for whole-grain cereals and T2D risk based on a few studies (n = 3 studies; RR 0.72 95% CI 0.55–0.93, *p* = 0.01). For each additional serving of whole-grain breakfast cereal RR 0.73, 95% CI: 0.59, 0.91; *p* = 0.006)	Ma1−
Chen et al. (2023) [73] PMID: 36854188	SR of three large prospective U.S. cohort studies	Nurses’ Health Study (n = 71,871) Nurses’ Health Study II (n = 87,918) Health Professional Follow-Up Study (n = 38,847)	Type 2 diabetes mellitus	Ultra-processed cereals were associated with lower T2D risk (HR 0.78; 95% CI 0.96–0.99). Among the ultra-processed breads and cereals further subdivision showed that intakes of ultra-processed cereals and ultra-processed dark breads and whole-grain breads were associated with lower T2D risk whilst ultra-processed refined breads were associated with higher risk.	Rs1+
Priebe and McMonagle et al. (2016) [1] PMID: 27749919	SR	6 RCTs (n = 130) 2 prospective studies included (n = 96,673)	Type 2 diabetes	Two studies showed that fibre-rich RTECs could benefit postprandial insulinemia other findings were less conclusive. Evidence from prospective studies suggests that whole-grain RTECs may have beneficial effects on the development of type 2 diabetes including positive effects of fibre-rich RTECs on postprandial insulinemia.	Rs1−
Williams et al. (2014) [25] PMID: 25225349	SR	3 of the 16 cohorts reported on breakfast cereal outcomes (n = 210,334)	Type 2 diabetes	Whole-grain or high-fibre breakfast cereals were associated with a lower risk of diabetes (evidence grade B).	Rs1−
Cardiovascular disease/lipid levels (7 studies)					
Sun et al. (2023) [74] PMID: 36803836	SR and dose–response MA of prospective studies	64 studies (for breakfast cereals 2 cohorts n = 453,632)	Cardiovascular disease mortality	There were protective associations for breakfast cereals and cardiovascular disease mortality (HR: 0.80; 95% CI: 0.70, 0.90).	Ma1++ Rs1++
Aune et al. (2016) [76] PMID: 27301975	Dose–response MA of prospective studies	45 cohort studies (64 publications) n = 4 for CHD n = 2 on CVD	Coronary heart disease Cardiovasular disease	Intake of whole-grain breakfast cereals and CHDs–high v low analysis (n = 4, RR 0.72, 95% CI 0.64–0.82, *p* = 0.92). Whole-grain breakfast cereals and CHDs (30 g/d dose, RR 0.81, 0.75–0.88, *p* = 0.69) Intake of whole-grain breakfast cereals and CVDs–high v low analysis (n = 2, RR 0.74, 95% CI 0.65–0.84, *p* = 0.31). Whole-grain breakfast cereals and CVDs (30 g/d dose, RR 0.84, 0.78–0.90, *p* = 0.82)	Ma1+
Kwok et al. (2019) [75] PMID: 30971126	MA	2 whole-grain breakfast cereal prospective studies (n = 206,200)	Cardiovascular disease and all-cause mortality	Among carbohydrates, there was a dose–response association for the benefit of whole-grain breakfast cereals (RR 0.84, 95% CI0.78–0.90, two studies).	Ma1−
Mendoza et al. (2024) [78] PMID: 39286398	SR of prospective cohorts	Nurses’ Health Study (n = 75,735) Nurses’ Health Study II (n = 90,813) Health Professionals Follow-Up Study (n = 40,409)	Cardiovascular disease and Stroke	Cold cereals were inversely associated with CVDs (HR 0.92), CHDs (HR 0.90) and stroke risk (HR 0.93). Foods in the bread/cold group included breakfast cereals; dark/whole-grain bread; refined-grain bread.	Rs1+CVD Rs1− Stroke
Beserra et al. (2020) [77] PMID: 33295516	SR	14 studies (n = 15,420)	Lipid profile of children and teens	Two out of fourteen studies found that increased intake of RTECs was related to reductions in total cholesterol and LDL-c.	Rs1−
Priebe MG and McMonagle JR (2016) [1] PMID: 27749919	SR of RCT and prospective studies	5 RCTs focused on blood lipids (n = 214) 3 prospective studies (n = 35,404)	Hypertension and blood lipids	Consumption of RTECs with soluble fibre helps to reduce LDL cholesterol in hypercholesterolemic men. Evidence from prospective studies suggests that whole-grain RTECs may have beneficial effects on hypertension. RTECs fortified with folate can reduce plasma homocysteine.	Rs1−
Williams et al. (2014) [25] PMID: 25225349	SR	5 cohort and case–control studies on breakfast cereals and CVDs (n = 139,552) 6 studies on breakfast cereal and hypertension (n = 28,467)	Health outcomes	Regular consumption of oat-, barley- or psyllium-based breakfast cereals can help lower total and LDL cholesterol concentrations (evidence grade A) Whole-grain or high-fibre breakfast cereals are associated with a lower risk of cardiovascular disease (evidence grade C).	Rs1−
Cancer (1 study)					
Aune et al. (2016) [76] PMID: 27301975	SR and dose–response MA of prospective studies	45 cohort studies (64 publications)	Cancer	Relative risks per 90 g/day increase in whole grain intake (90 g equivalent to one bowl of cereal was associated with a reduced risk of total cancer 0.83 (0.77 to 0.90; I(2) = 83%, n = 11). Note: This finding is for 90 g increase in whole grain in general, which included whole-grain breakfast cereals, whole-grain bread, added bran, total bread and breakfast cereals.	Rs/Ma1+

Note: As there were so many studies, this search was restricted to SRs and MAs. Key: BMI, body mass index; CI, confidence interval; DF, dietary fibre; GI, glycaemic index; HR, hazard ratio; LDL, low-density lipoprotein; RR, relative risk; RTE, ready-to-eat cereal; SDS, slowly digestible starch.

### Appendix B.6. Studies Investigating Subgroups of UPFs Including RTEC and Health Outcomes

**Table:**

Author, Year, PMID	Country	Study Design	Number of Studies (Number of Participants)	Results	Level of Evidence
Taneri et al. (2022) [80] PMID: 35231930	NA	SR and MA	40 prospective cohort studies (n = 5750,133)	Breakfast cereals were associated with a lower mortality risk (RR = 0.85, 95% CI, 0.79, 0.92).	Rs/Ma1++
Mendoza et al. (2024) [78] PMID: 39286398	US	SR and MA of three prospective cohorts	Nurses’ Health Study (n = 75,735) Nurses’ Health Study II (n = 90,813) Health Professionals Follow-Up Study (n = 40,409)	Colds cereals were inversely associated with CVD (HR 0.92), CHD (HR 0.90) and stroke risk (HR 0.93).	Rs1+CVD Rs1− Stroke
Chen et al. (2023) [73] PMID: 36854188	US	MA of three large prospective U.S. cohort studies	Nurses’ Health Study (n = 71,871) Nurses’ Health Study II (n = 87,918) Health Professional Follow-Up Study (n = 38,847)	Ultra-processed cereals were associated with lower T2D risk (HR 0.78).	Rs1+
Beserra et al. (2020) [77] PMID: 33295516	NA	SR of cross-sectional and longitudinal studies, with or without intervention	14 studies (n = 15,420)	Of the 14 studies included, 9 demonstrated that ultra-processed food consumption was related to increased LDL-c, total cholesterol, triglycerides and a reduction in HDL-c. Three studies found no relationship. Two found that increased intake of RTECs was related to reductions in total cholesterol and LDL-c.	Rs1+
Dicken et al. (2024) [81] PMID: NA	Europe	Prospective cohort analysis of EPIC	n = 311,892 n = 14,236 T2DM cases	Breakfast cereals, breads and biscuits (as a group) were associated with lower-incident type 2 diabetes mellitus (HR 0.65, 0.57–0.73) for each 10%g/day increase in the diet	Coh2−
Cordova et al. (2023) [24] PMID: 38115963	Europe	Multinational EPIC cohort	n = 266,666 (60% women)	The subgroup of ultra-processed breads and cereals (HR: 0.97, 95% CI: 0.94, 1.00) was not associated with risk of cancer and cardiometabolic diseases.	Coh2+
Hang et al. (2023) [82] PMID: 36477589	US	3 prospective cohorts	n = 142 052	High-risk polyps were associated with ultra-processed breads and breakfast foods (HR: 1.13, 95% CI = 1.03 to 1.24). This category included breakfast bars, cold breakfast cereal, English muffins, bagels, rolls, rye, pumpernickel bread, white bread, and whole-grain bread.	Coh2+
Lo et al. (2022) [83] PMID: 34461300	US	Prospective cohort study	n = 245,112	Ultra-processed breads and breakfast foods were more strongly associated with Crohn’s Disease (HR: 1.18, 95% CI 1.07–1.29). This category included cold breakfast cereal, English muffins, bagels, and rolls, white bread.	Coh2−
Belchor et al. (2022) [84] PMID: 36317890	Brazil	Cross-sectional study	n = 1069 students in Florianopolis, southern Brazil	Breakfast cereal consumption was inversely assoc. with the ultra-processed dietary profile (−0.070; Kaiser–Meyer–Olkin statistical test).	NA

Key: CI, confidence interval; CHD, coronary heart disease; CVD, cardiovascular disease; EPIC, European Prospective Investigation into Cancer and Nutrition; HR, hazard ratio; LDL-c, low-density lipoprotein cholesterol; RR, relative risk; RTE, ready-to-eat; T2DM, type 2 diabetes mellitus; UPF, ultra-processed food.
